# High dose IFN-α2b as post operative adjuvant therapy for acral melanoma: a multi-center real-world cohort study of 171 Chinese patients

**DOI:** 10.3389/fonc.2026.1886308

**Published:** 2026-08-17

**Authors:** Kaidi Yang, Shiji Lan, Yan Wang, Jin Wu, Xin Cai, Kui Jiang, Zhen Guo, Mei Xiang, Hongfeng Zhou, Jiwei Li, Fang Liu, Jianping Gui, Yuhuan Mao, Hongxue Meng, Xiang Ji, Janan Pang, Xinxin Yang, Lu Feng, Weizhen Zhang, Di Wu

**Affiliations:** 1 Cancer Center, The First Hospital of Jilin University, Changchun, China; 2 Medical Oncology, The Third People’s Hospital of Zhengzhou, Zhengzhou, China; 3 Department of head and neck genitourinary medicine, Harbin Medical University Cancer Hospital, Harbin, China; 4 The First Affiliated Hospital of Dalian Medical University, Dalian, China; 5 Department of Medical Oncology, The Second Affiliated Hospital of Dalian Medical University, Dalian, China

**Keywords:** acral melanoma, adjuvant therapy, clinical and pathological characteristics, high-dose interferon-alpha-2b, immunotherapy

## Abstract

**Background:**

This study aimed to investigate recurrence patterns and survival outcomes in Chinese patients with AM who received postoperative adjuvant High-dose interferon (HD-IFN), and to assess how treatment duration and the interval between surgery and therapy initiation relate to recurrence and survival.

**Methods:**

A multi-center, retrospective cohort study involving 171 patients with AM who received postoperative adjuvant HD-IFN therapy between January 2012 and March 2025 was conducted at five melanoma diagnostic and treatment centers across China.

**Results:**

The median follow-up duration was 75.7 months. Median recurrence-free survival (RFS) and overall survival (OS) were 37.5 and 93.7 months, respectively. Stratified by recurrence risk, patients in the low-risk, high-risk, and very high-risk groups had median RFS of 64.1, 31.6, and 13.8 months (*P* = 0.035), and median OS of 131.9, 82.1, and 61.9 months, respectively (*P* = 0.027).

**Conclusion:**

Results show that full-course HD-IFN treatment is statistically associated with improved survival in high-risk and very high-risk subgroups, whereas the timing of HD-IFN initiation within 3 months after surgery makes no significant difference to prognosis.

## 1 Introduction

Melanoma is a malignant tumor originating from melanocytes. Acral melanoma (AM) is the most prevalent subtype among Asian populations, accounting for 47.5-65% of all melanomas, in contrast to the cutaneous form, which predominates in Caucasian populations (1–4). AM typically arises on the palms, soles, and nail beds, and is associated with poorer prognosis compared to other melanoma subtypes, largely due to its distinct biological behaviour (5–12).

High-dose interferon-alpha-2b (HD-IFN) was the first systemic adjuvant therapy approved by the United States Food and Drug Administration (FDA) for melanoma, based on evidence from several large-scale prospective randomized controlled trials conducted in the late 20th century (13–15). With the advent of targeted therapies (e.g., BRAF inhibitors) and immune checkpoint inhibitors (ICIs), these newer agents have largely replaced HD-IFN in the adjuvant treatment of cutaneous melanoma (CM) (16, 17). However, HD-IFN continues to play a central role in the adjuvant management of AM, particularly in patients with tumors lacking BRAF mutations and those who exhibit limited response to ICIs (18).

Despite its clinical relevance, few large-scale studies have evaluated the use of HD-IFN in AM. Although some prospective trials have been conducted, their stringent eligibility criteria limit generalizability and do not adequately reflect real-world, patient-centered outcomes. Additionally, several important clinical questions remain unresolved. For example, it is still unclear how the duration of HD-IFN adjuvant therapy influences treatment efficacy in AM. Similarly, the timing of therapy initiation following surgery also remains a critical factor to be determined.

Therefore, a real-world retrospective study was conducted to evaluate the outcomes of Chinese patients with AM who received postoperative adjuvant HD-IFN. The impact of the duration of intensive-phase HD-IFN treatment and the interval between surgery and treatment initiation on postoperative recurrence and survival were specifically investigated in this study, thereby providing evidence-based guidance for optimizing treatment strategies and improving clinical outcomes in patients with AM.

## 2 Patients and methods

### 2.1 Patients

This multi-center retrospective study included 845 consecutive patients with AM treated between January 2012 and March 2025 at five melanoma treatment centers in China. A total of 171 patients were enrolled for subsequent analysis after screening (**Supplementary Figure 1**).

Eligible patients met the following inclusion criteria: (1) histologically confirmed stage I–III primary AM that had been completely resected with clear surgical margins before subgroup assignment; (2) normal function of all major organs and bone marrow, with no prior systemic adjuvant therapy; (3) no evidence of distant metastasis confirmed by lymph node ultrasound, whole-body helical computed tomography (CT), or positron emission tomography–CT; and (4) an Eastern Cooperative Oncology Group (ECOG) performance status of 0 or 1. Exclusion criteria were: (1) patients with stage IV AM following radical resection; (2) patients who received no postoperative adjuvant therapy or adjuvant regimens other than HD-IFN; (3) patients with incomplete follow-up or medical record data; and (4) patients with a second primary tumor. Ultimately, 171 patients were included in the final analysis. This study was approved by the Ethics Committee of the First Hospital of Jilin University (AF-IRB-032-06), and ethical approvals were obtained from all participating centers.

### 2.2 Risk stratification

Among the 171 AM patients, recurrence and survival outcomes were analyzed in the total cohort, across different disease stages, and within stratified risk groups. Of these patients, 2 had stage 0 disease, 12 had stage IA, 3 had stage I, 3 had stage II, and 5 had stage III. In this study, risk stratification was performed in accordance with the AJCC 8th Edition melanoma staging system and the Chinese Consensus on the Diagnosis and Treatment of Acral Melanoma. Patients were divided into three groups—low-risk (stages IB–IIA, n = 63), high-risk (stages IIB–IIIA, n = 63), and very high-risk (stages IIIB–IIID, n = 20)—for further subgroup analysis.

### 2.3 Information on HD-IFN adjuvant therapy

A total of 171 patients with acral melanoma receiving postoperative adjuvant HD-IFN therapy were enrolled. Baseline risk stratification yielded 63 patients in the low-risk group, 63 in the high-risk group and 20 in the ultrahigh-risk group. Among these, 11 low-risk and 16 high-risk patients failed to complete the 4-week intensive induction phase. These patients were only included in baseline characteristic descriptions and overall survival analyses, and excluded from subsequent subgroup analyses regarding treatment duration. In the cohort used for stratified analysis, there were 52 remaining cases in the low-risk group, 47 in the high-risk group, and 20 in the very high-risk group. In the valid cohort used for stratified analysis, the low-risk group consisted of 52 remaining cases, which were divided into three subgroups: A1, A2, and A3. The high-risk group consisted of 47 cases, and the very high-risk group consisted of 20 cases, which were divided into two subgroups, A3 and A4, for analysis.

### 2.4 HD-IFN dosing groups and timing

To evaluate the impact of HD-IFN duration on prognosis, patients were categorized based on the length of their adjuvant interferon treatment. Due to the limited number of patients who completed the full 1-year HD-IFN course, the X-tile software was used to determine an optimal cut-off point of 160 days for treatment duration. Based on this, patients were classified into four groups: (1) Group A1: completed the 4-week intensive regimen (18–20 MIU, five days per week); (2) Group A2: completed the 4-week regimen, followed by a maintenance course (<160 days of 9–10 MIU, three times weekly); (3) Group A3: completed the 4-week regimen, followed by ≥160 days of maintenance treatment; (4) Group A4: included all patients from Group A1 and A2 combined.

To assess the effect of initiation timing, the interval between surgery and the first dose of HD-IFN (denoted as *T*) was recorded. Patients were stratified into two groups based on this interval: (1) Group B1: *T* between 0 and 30 days; (2) Group B2: *T* between 31 and 90 days.

Subgroup analyses by risk category were as follows: (1) For the low-risk group (stage IB–IIA, n = 52), patients were divided into Groups A1, A2, and A3; (2) For the high-risk (stage II–IIIA, n = 47) and very high-risk (stage IIIB–IIID, n = 20) groups, analyses were conducted using Groups A3 and A4.

### 2.5 Study endpoints

The primary endpoints were recurrence-free survival (RFS) and overall survival (OS). All patients were staged according to the 8th edition of the American Joint Committee on Cancer (AJCC) Melanoma Staging System.

### 2.6 Statistical analysis

Survival curves were estimated using the Kaplan-Meier method, and differences between groups were evaluated using the log-rank test. Assess risk factors for PFS and OS using the COX proportional hazards model. Using X-tile software with RFS as the endpoint, we performed an exhaustive search within the study dataset to identify the optimal cutoff value for the duration of HD-IFN treatment, ultimately determining the cutoff point to be 160 days. This cutoff value was derived from this cohort’s data; no independent validation cohort was established, and no multiple-test correction was performed for the multiple cutoff-point searches, which may pose a risk of overfitting.

This study recorded the completion status of HD-IFN treatment, reasons for discontinuation, and adverse reactions of all grades in accordance with the CTCAE 5.0 criteria, and compared toxicity incidence rates by grouping patients according to risk stratification and treatment cycles. The χ² test was used for comparisons of incidence rates across multiple groups; when theoretical frequencies were insufficient, Fisher’s exact test using an R×C Table was employed. If the overall difference was statistically significant, pairwise comparisons were conducted after Bonferroni correction.

RFS was defined as the time from diagnosis to the first instance of local, regional, or distant recurrence. For patients without recurrence, RFS was censored at the date of death, the last recurrence-free follow-up, or the end of the study period—whichever occurred first. OS was defined as the time from diagnosis to death from any cause. For patients alive at the study’s end, OS was censored at the time of the last follow-up. For patients lost to follow-up, both RFS and OS were censored at the date of last known recurrence-free or survival status, respectively. Continuous variables were converted into categorical variables for analysis. A two-sided *p* value < 0.05 was considered statistically significant. All statistical analyses were performed using SPSS software, version 27.0 (IBM Corp.).

## 3 Results

### 3.1 Patient characteristics and prognostic outcomes

**Figure 1:**
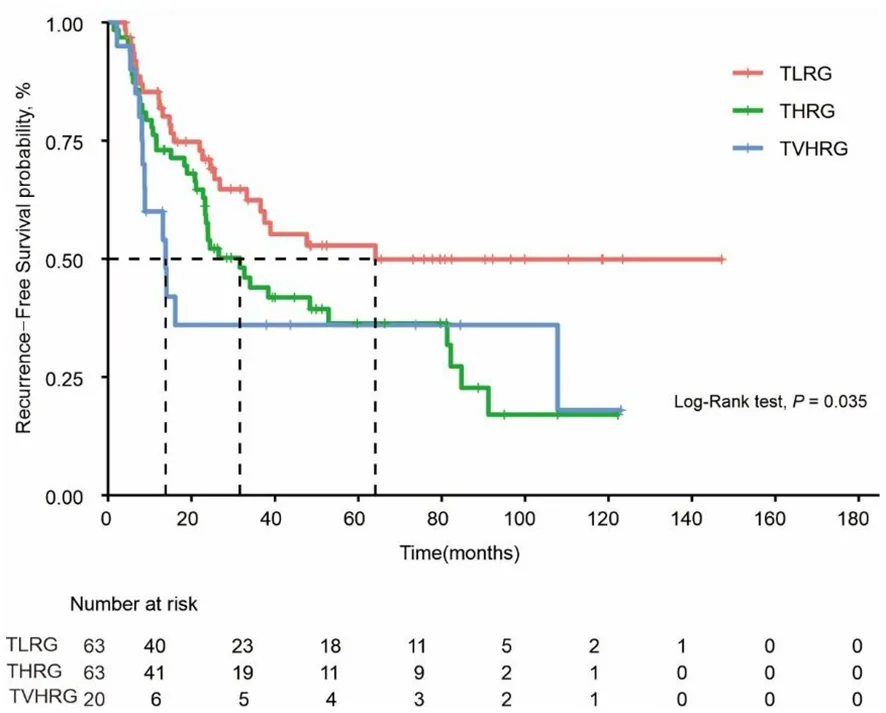
RFS of patients stratified into low-risk, high-risk and very-high-risk groups. RFS, relapse-free survival; TLRG, the low-risk group; THRG, the high-risk group; TVHRG, the very high-risk group.

**Figure 2:**
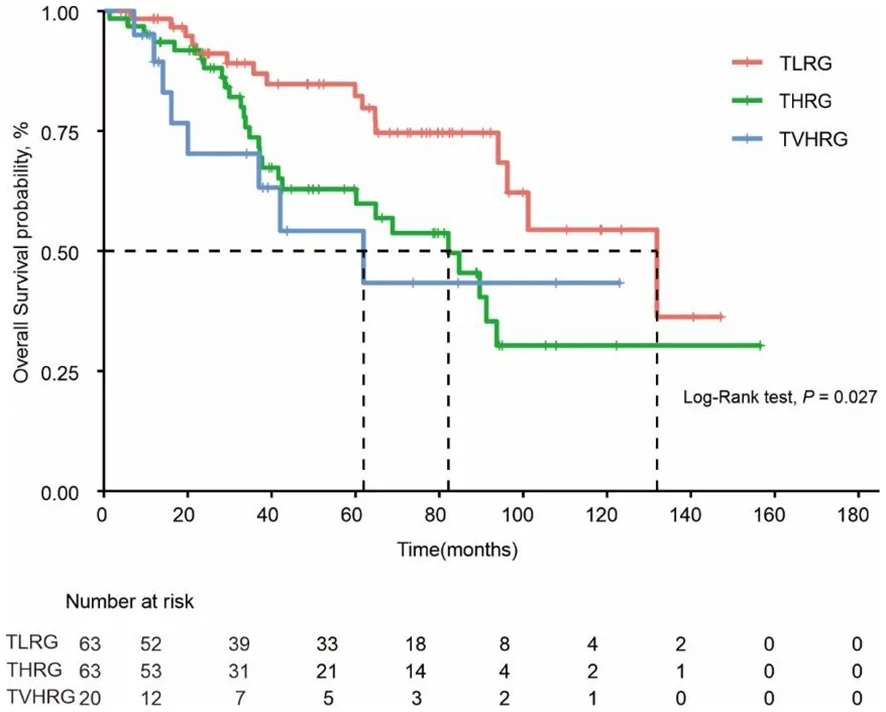
OS of patients stratified into low-risk, high-risk and very-high-risk groups. OS, overall survival; TLRG, the low-risk group; THRG, the high-risk group; TVHRG, the very high-risk group.

A total of 171 patients with AM who received HD-IFN as postoperative adjuvant therapy were included (**Table 1**). The cohort comprised slightly more male than female patients (50.9% vs. 49.1%), with a median age of 57 years. Most primary tumors were located on the soles (68.4%), followed by the palms (21.0%) and nail beds (10.5%). The median follow-up duration was 75.8 months. Patients were stratified by recurrence risk into three groups: low-risk (n = 63), high-risk (n = 63), and very high-risk (n = 20). Detailed baseline clinical data for the three groups are presented in **Tables 2**–**4**. The median RFS for the three groups was 64.1, 31.6, and 13.8 months, respectively (*P* = 0.035), and the median OS was 131.9, 82.2, and 61.9 months, respectively (*P* = 0.027) (**Figures 1**, **2**).

**Table 1:** Clinicopathological characteristics of the total population.

Variables, n (%)		Total (n=171)
Gender		
	Male	84 (49.13)
	Female	87 (50.87)
Age, y		
	<60	103 (60.24)
	≥60	68 (39.76)
Primary site		
	Palm	36 (21.05)
	Sole	116 (67.84)
	Nail	19 (11.11)
Stage		
	0-IA	14 (8.19)
	IB	25 (14.62)
	IIA	38 (22.22)
	IIB	36 (21.05)
	IIC	26 (15.20)
	IIIA	1 (0.58)
	IIIB	9 (5.26)
	IIIC	8 (4.68)
	IIID	3 (1.76)
	Unknown	11 (6.44)
Mitotic rate/mm2		
	<1	8 (4.68)
	≥1	126 (73.68)
	Unknown	37 (21.64)
Ulceration		
	Yes	92 (53.80)
	No	68 (39.76)
	Unknown	11 (6.44)
Breslow thickness, mm		
	≤1-4	108 (63.17)
	>4	52 (30.40)
	Unknown	11 (6.43)
Clark level		
	I/II	18 (10.52)
	III	17 (9.94)
	IV	52 (30.41)
	V	17 (9.95)
	Unknown	67 (39.18)
Lymphovascular invasion		
	Yes	8 (4.67)
	No	146 (85.38)
	Unknown	17 (9.95)
Neurotropism		
	Yes	4 (2.34)
	No	147 (85.97)
	Unknown	20 (11.69)
Microsatellite		
	Yes	2 (1.17)
	No	118 (69.01)
	Unknown	51 (29.82)
Ki-67, %		
	≤30	73 (42.69)
	>30	25 (14.62)
	Unknown	73 (42.69)
Trauma		
	Yes	56 (32.75)
	No	105 (61.41)
	Unknown	10 (5.84)
SLNB		
	Yes	63 (36.84)
	No	108 (63.16)
SLNB status		
	Positive	9 (5.27)
	Negative	54 (31.58)
	Unknown	108 (63.15)

SLNB, sentinel lymph node biops.

**Table 2:** Clinicopathological characteristics of the low-risk group.

Variables, n (%)		The low-risk (n=63)
Gender		
	Male	25 (39.68)
	Female	38 (60.32)
Age, y		
	<60	42 (66.67)
	≥60	21 (33.33)
Primary site		
	Palm	15 (23.82)
	Sole	41 (65.07)
	Nail	7 (11.11)
Stage		
	0-IA	0 (0)
	IB	25 (39.68)
	IIA	38 (60.32)
	IIB	0 (0)
	IIC	0 (0)
	IIIA	0 (0)
	IIIB	0 (0)
	IIIC	0 (0)
	IIID	0 (0)
	Unknown	0 (0)
Mitotic rate/mm2		
	<1	5 (7.94)
	≥1	51 (80.95)
	Unknown	7 (11.11)
Ulceration		
	Yes	19 (30.15)
	No	41 (65.08)
	Unknown	3 (4.77)
Breslow thickness, mm		
	≤1-4	5 (85.72)
	>4	8 (12.69)
	Unknown	1 (1.59)
Clark level		
	I/II	8 (12.69)
	III	12 (19.05)
	IV	19 (30.16)
	V	2 (3.18)
	Unknown	22 (34.92)
Lymphovascular invasion		
	Yes	1 (1.58)
	No	59 (93.66)
	Unknown	3 (4.76)
Neurotropism		
	Yes	2 (3.17)
	No	54 (85.72)
	Unknown	7 (11.11)
Microsatellite		
	Yes	0 (0)
	No	43 (68.25)
	Unknown	20 (31.75)
Ki-67, %		
	≤30	32 (50.79)
	>30	6 (9.53)
	Unknown	25 (39.68)
Trauma		
	Yes	22 (34.92)
	No	37 (58.73)
	Unknown	4 (6.35)
SLNB		
	Yes	18 (28.57)
	No	45 (71.43)
SLNB status		
	Positive	0 (0.0)
	Negative	18 (28.57)
	Unknown	45 (71.43)

The high-risk: stage IB-IIA.

SLNB, sentinel lymph node biops.

**Table 3:** Clinicopathological characteristics of the high-risk group.

Variables, n (%)		The high-risk (n=63)
Gender		
	Male	32 (50.79)
	Female	31 (49.21)
Age, y		
	<60	35 (55.56)
	≥60	28 (44.44)
Primary site		
	Palm	13 (20.64)
	Sole	39 (61.90)
	Nail	11 (17.46)
Stage		
	0-IA	0 (0)
	IB	0 (0)
	IIA	0 (0)
	IIB	36 (57.15)
	IIC	26 (41.26)
	IIIA	1 (1.59)
	IIIB	0 (0)
	IIIC	0 (0)
	IIID	0 (0)
	Unknown	0 (0)
Mitotic rate/mm2		
	<1	3 (4.76)
	≥1	45 (71.43)
	Unknown	15 (23.81)
Ulceration		
	Yes	52 (82.54)
	No	9 (14.29)
	Unknown	2 (3.17)
Breslow thickness, mm		
	≤1-4	22 (34.92)
	>4	40 (63.49)
	Unknown	1 (1.59)
Clark level		
	I/II	1 (1.59)
	III	4 (6.35)
	IV	24 (38.09)
	V	9 (14.29)
	Unknown	25 (39.68)
Lymphovascular invasion		
	Yes	4 (6.35)
	No	49 (77.78)
	Unknown	10 (15.87)
Neurotropism		
	Yes	1 (1.59)
	No	54 (85.72)
	Unknown	8 (12.69)
Microsatellite		
	Yes	1 (1.59)
	No	43 (68.25)
	Unknown	19 (30.16)
Ki-67, %		
	≤30	25 (39.68)
	>30	14 (22.22)
	Unknown	24 (38.10)
Trauma		
	Yes	23 (36.50)
	No	35 (55.56)
	Unknown	5 (7.94)
SLNB		
	Yes	25 (39.68)
	No	38 (60.32)
SLNB status		
	Positive	2 (3.18)
	Negative	23 (36.50)
	Unknown	38 (60.32)

The high-risk: stage IIB-IIIA.

SLNB, sentinel lymph node biops .

**Table 4:** Clinicopathological characteristics of the very high-risk group.

Variables, n (%)		The very high-risk (n=20)
Gender		
	Male	12 (60.00)
	Female	8 (40.00)
Age, y		
	<60	10 (50.0)
	≥60	10 (50.0)
Primary site		
	Palm	3 (15.00)
	Sole	16 (80.00)
	Nail	1 (5.00)
Stage		
	0-IA	0 (0)
	IB	0 (0)
	IIA	0 (0)
	IIB	0 (0)
	IIC	0 (0)
	IIIA	0 (0)
	IIIB	9 (45.00)
	IIIC	8 (40.00)
	IIID	3 (15.00)
	Unknown	0 (0)
Mitotic rate/mm2		
	<1	1 (5.00)
	≥1	17 (85.00)
	Unknown	2 (10.00)
Ulceration		
	Yes	13 (65.00)
	No	6 (30.00)
	Unknown	1 (5.00)
Breslow thickness, mm		
	≤1-4	11 (55.00)
	>4	9 (45.00)
	Unknown	0 (0)
Clark level		
	I/II	1 (5.00)
	III	0 (0)
	IV	7 (35.00)
	V	5 (25.00)
	Unknown	7 (35.00)
Lymphovascular invasion		
	Yes	3 (15.00)
	No	16 (80.00)
	Unknown	1 (5.00)
Neurotropism		
	Yes	1 (5.00)
	No	17 (85.00)
	Unknown	2 (10.00)
Microsatellite		
	Yes	1 (5.00)
	No	13 (65.00)
	Unknown	6 (30.00)
Ki-67, %		
	≤30	12 (60.00)
	>30	3 (15.00)
	Unknown	5 (25.00)
Trauma		
	Yes	6 (30.00)
	No	13 (65.00)
	Unknown	1 (5.00)
SLNB		
	Yes	6 (30.00)
	No	14 (70.00)
SLNB status		
	Positive	5 (25.00)
	Negative	1 (5.00)
	Unknown	14 (70.00)

The very high-risk: stage IIIB-IIID.

SLNB, sentinel lymph node biops.

### 3.2 Analysis of RFS, OS, and prognostic factors

#### 3.2.1 Analysis of RFS and prognostic factors in the total population

**Figure 3:**
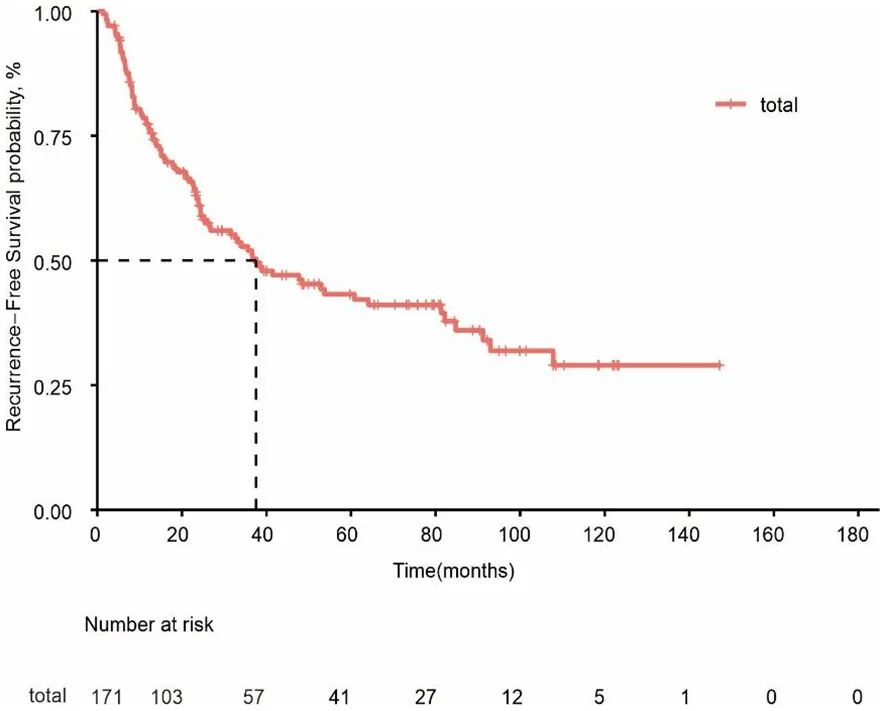
Kaplan-Meier analysis of RFS in the total population of AM patients. RFS, Relapse-Free Survival; AM, Acral Melanoma.

A Kaplan-Meier survival analysis was performed on 171 patients; the 1-, 3-, and 5-year RFS rates were 77.3%, 52.0%, and 43.2%, respectively, with a median RFS of 37.5 months (95% CI 21.8–53.2) (**Figure 3**).

**Figure 4:**
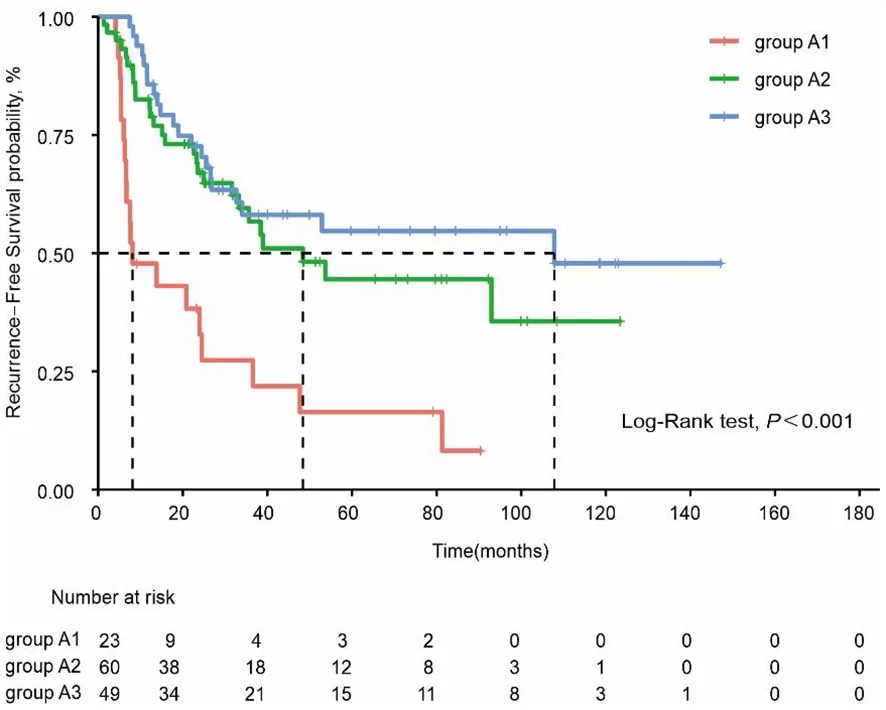
Kaplan-Meier analysis of RFS stratified by HD-IFN treatment duration in the total population of AM patients. RFS, Relapse-Free Survival; HD-IFN, High-dose interferon-alpha-2b; AM, Acral Melanoma.

**Figure 5:**
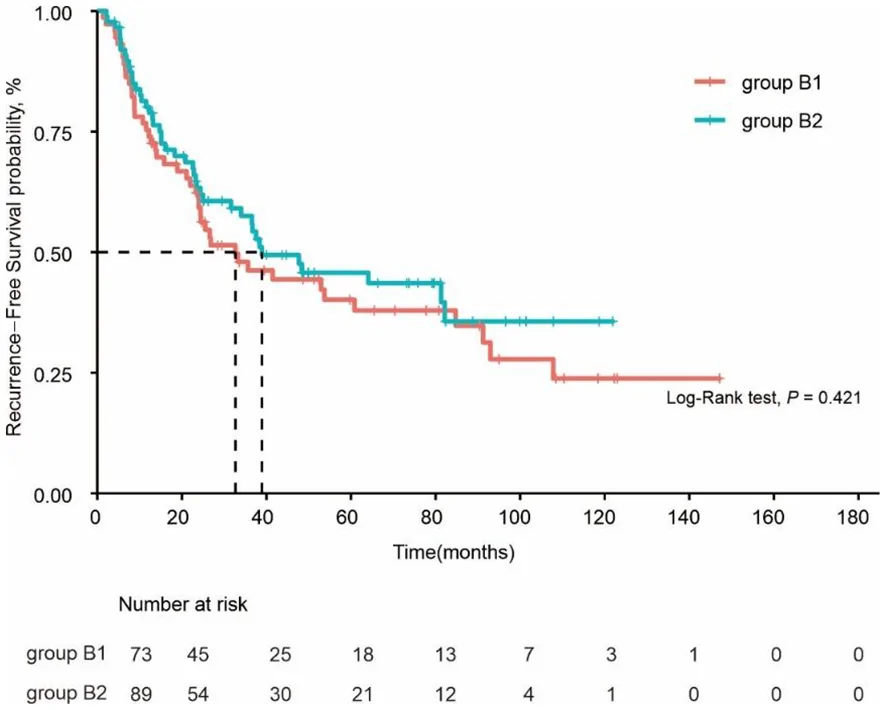
Kaplan-Meier analysis of RFS stratified by the interval from surgery to initial HD-IFN administration in the total population of AM patients. RFS, Relapse-Free Survival; HD-IFN, High-dose interferon-alpha-2b; AM, Acral Melanoma.

The 5-year RFS rates for patients classified into treatment regimens A1, A2, and A3 based on HD-IFN were 46.9%, 15.3%, and 54.7%, respectively (*P* < 0.001) (**Figure 4**). There was no statistically significant difference in median RFS between patients who initiated treatment within 30 days postoperatively and those who initiated treatment between 31 and 90 days postoperatively (*P* = 0.421) (**Figure 5**). Patients under 60 years of age (*P* = 0.007) and those without ulceration at the primary site (*P* = 0.006) had better RFS. Gender, mitotic count, and history of trauma were not significantly associated with RFS.

Univariate Cox analysis indicated that duration of HD-IFN therapy, age, and the presence ofulcers were associated with RFS; multivariate regression confirmed that duration of HD-IFN therapy is an independent prognostic factor for RFS (**Supplementary Table 1**).

#### 3.2.2 Analysis of OS and prognostic factors in the total population

**Figure 6:**
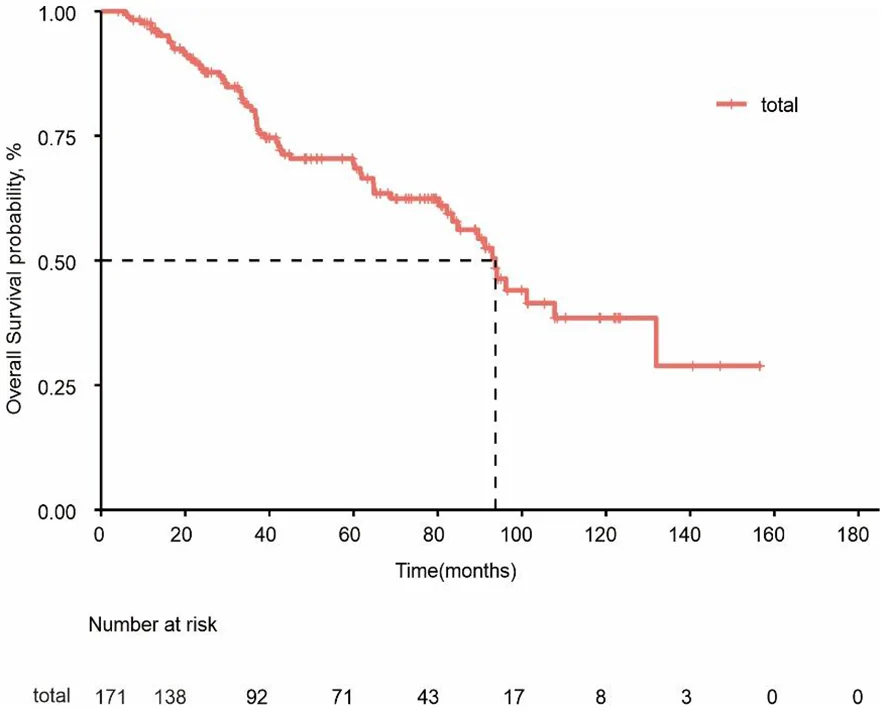
Kaplan-Meier analysis of OS in the total population of AM patients. OS, Overall Survival; AM, Acral Melanoma.

The 1-, 3-, and 5-year OS rates were 96.4%, 80.1%, and 69.4%, respectively, with a median OS of 93.7 months (95% CI 83.3–104.0) (**Figure 6**).

**Figure 7:**
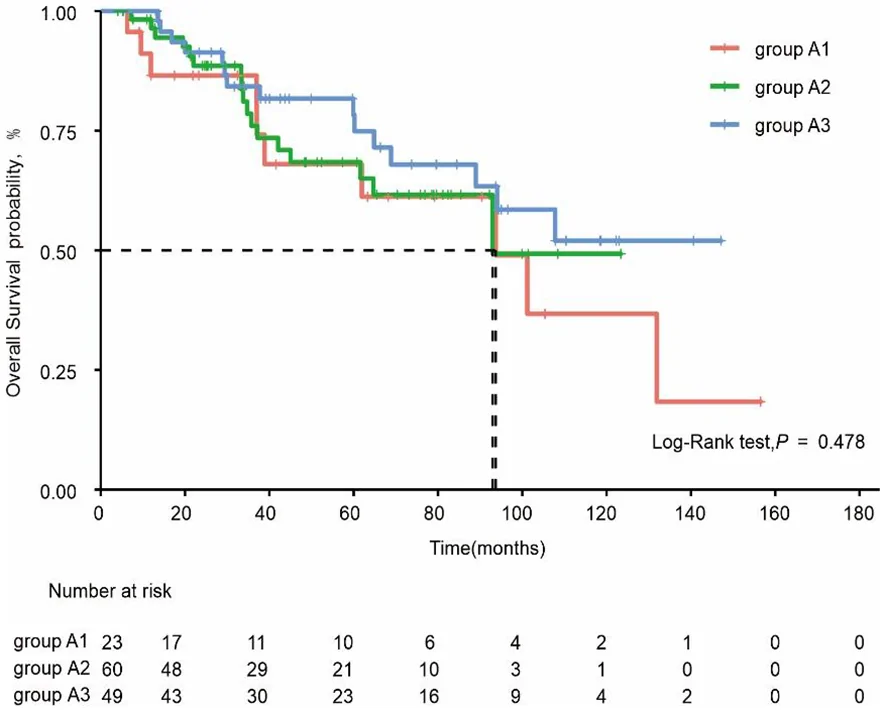
Kaplan-Meier analysis of OS stratified by HD-IFN treatment duration in the total population of AM patients. OS, Overall Survival; HD-IFN, High-dose interferon-alpha-2b; AM, Acral Melanoma.

**Figure 8:**
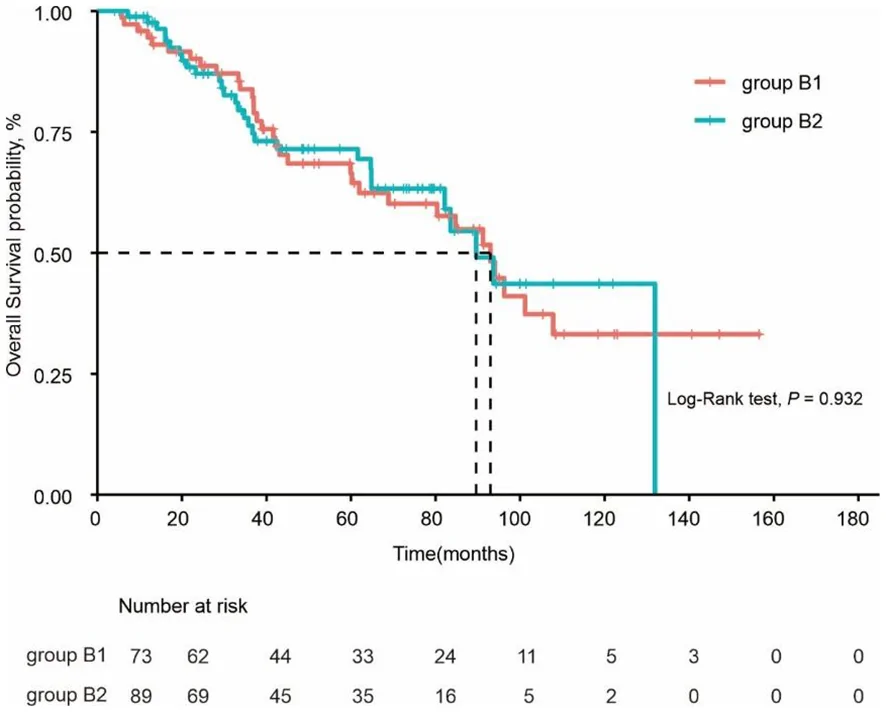
Kaplan-Meier analysis of OS stratified by the interval from surgery to initial HD-IFN administration in the total population of AM patients. OS, Overall Survival; HD-IFN, High-dose interferon-alpha-2b; AM, Acral Melanoma.

The 5-year OS rates for groups A1, A2, and A3 were 67.7%, 69.7%, and 78.3%, respectively (*P* = 0.478) (**Figure 7**). The median OS for groups B1 and B2 were 92.9 months and 89.6 months, respectively (*P* = 0.932) (**Figure 8**). Patients under 60 years of age (*P* = 0.032) and those without ulcers (*P* = 0.007) had a better OS prognosis. Gender, mitotic count, and trauma were not significantly associated with OS.

In univariate analysis, age and ulcers were associated with OS. However, multivariate analysis did not identify any independent risk factors for OS (**Supplementary Table 2**).

#### 3.2.3 Analysis of RFS and prognostic factors in the low-risk group

**Figure 9:**
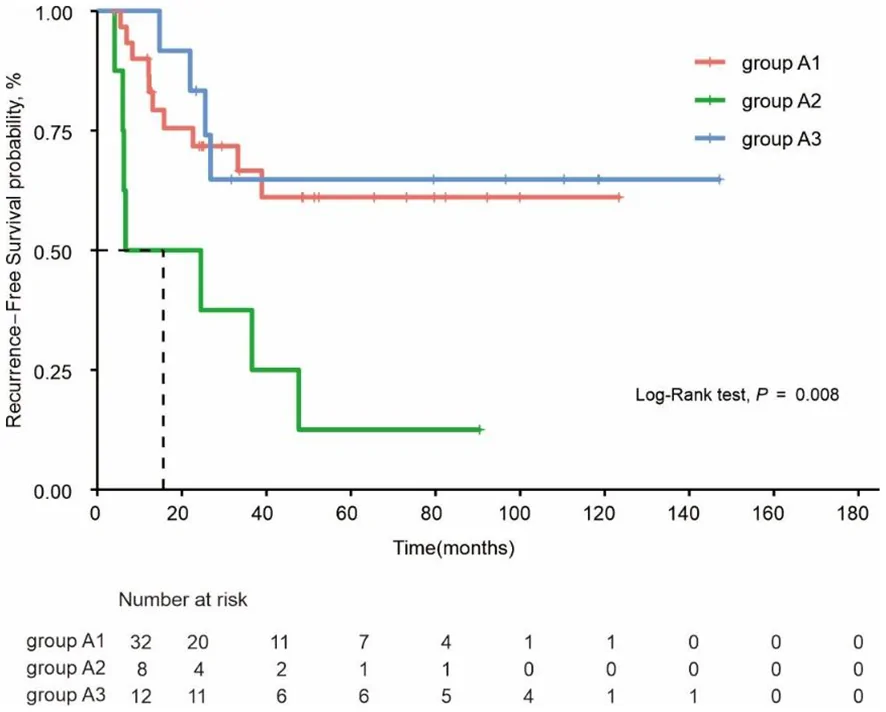
Kaplan-Meier analysis of RFS stratified by HD-IFN treatment duration in the low-risk group of AM patients. RFS, Relapse-Free Survival; HD-IFN, High-dose interferon-alpha-2b; AM, Acral Melanoma.

**Figure 10:**
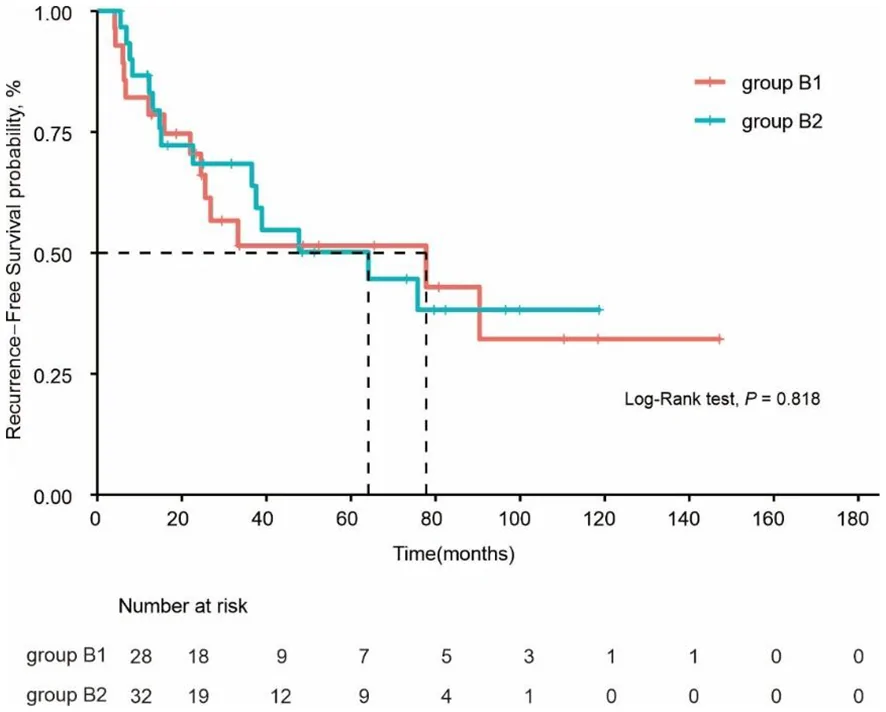
Kaplan-Meier analysis of RFS stratified by the interval from surgery to initial HD-IFN administration in the low-risk group of AM patients. RFS, Relapse-Free Survival; HD-IFN, High-dose interferon-alpha-2b; AM, Acral Melanoma.

The 5-year RFS rates for groups A1, A2, and A3 were 61.1%, 12.5%, and 64.8%, respectively, with a statistically significant difference between groups (*P* = 0.008) (**Figure 9**). The median RFS for groups B1 and B2 were 77.8 months and 64.1 months, respectively (*P* = 0.818) (**Figure 10**). Age, gender, presence or absence of ulceration, mitotic rate, and history of trauma were not significantly associated with RFS.

The results of the univariate analysis indicate that the duration of IFN treatment is statistically associated with RFS in AM patients in the low-risk group (**Supplementary Table 3**).

#### 3.2.4 Analysis of OS and prognostic factors in the low-risk group

**Figure 11:**
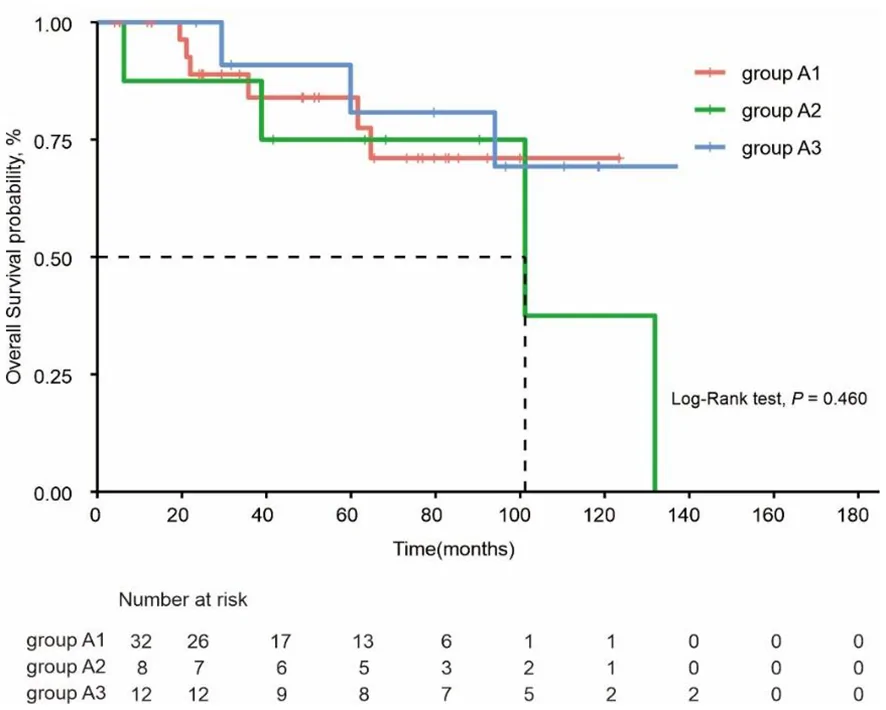
Kaplan-Meier analysis of OS stratified by HD-IFN treatment duration in the low-risk group of AM patients. OS, Overall Survival; HD-IFN, High-dose interferon-alpha-2b; AM, Acral Melanoma.

**Figure 12:**
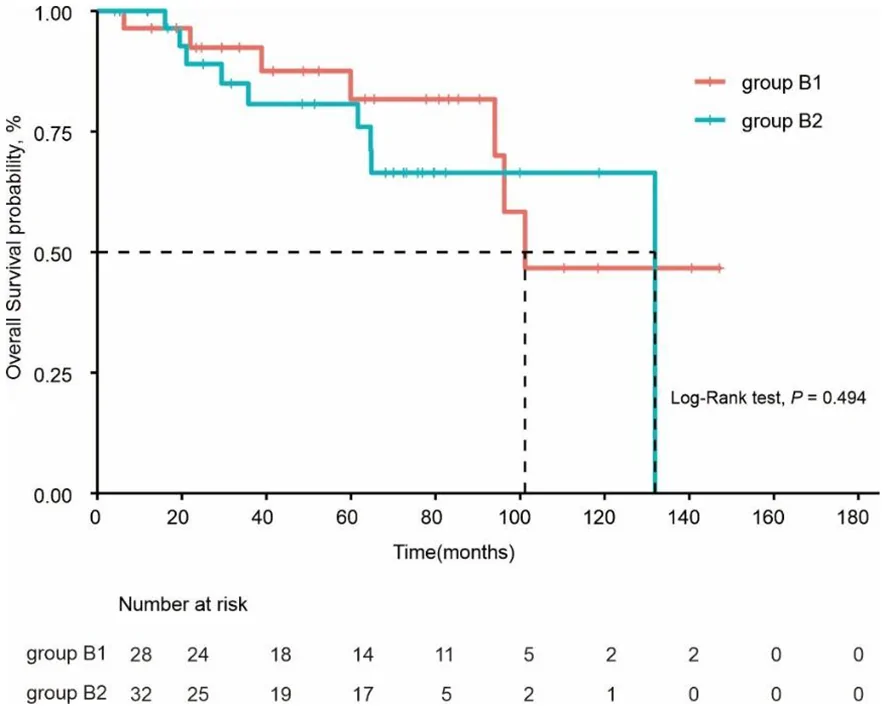
Kaplan-Meier analysis of OS stratified by the interval from surgery to initial HD-IFN administration in the low-risk group of AM patients. OS, Overall Survival; HD-IFN, High-dose interferon-alpha-2b; AM, Acral Melanoma.

The 5-year OS rates for groups A1, A2, and A3 were 84.0%, 75.0%, and 80.8%, respectively (*P* = 0.460) (**Figure 11**). The median OS for groups B1 and B2 were 101.2 months and 131.9 months, respectively (*P* = 0.494) (**Figure 12**). No significant associations were found between OS and age, gender, presence or absence of ulceration, mitotic rate, or history of trauma.

Univariate analysis showed that none of the variables were significantly associated with OS, and no independent prognostic factors were identified (**Supplementary Table 4**).

#### 3.2.5 Analysis of RFS and prognostic factors in the high-risk group

**Figure 13:**
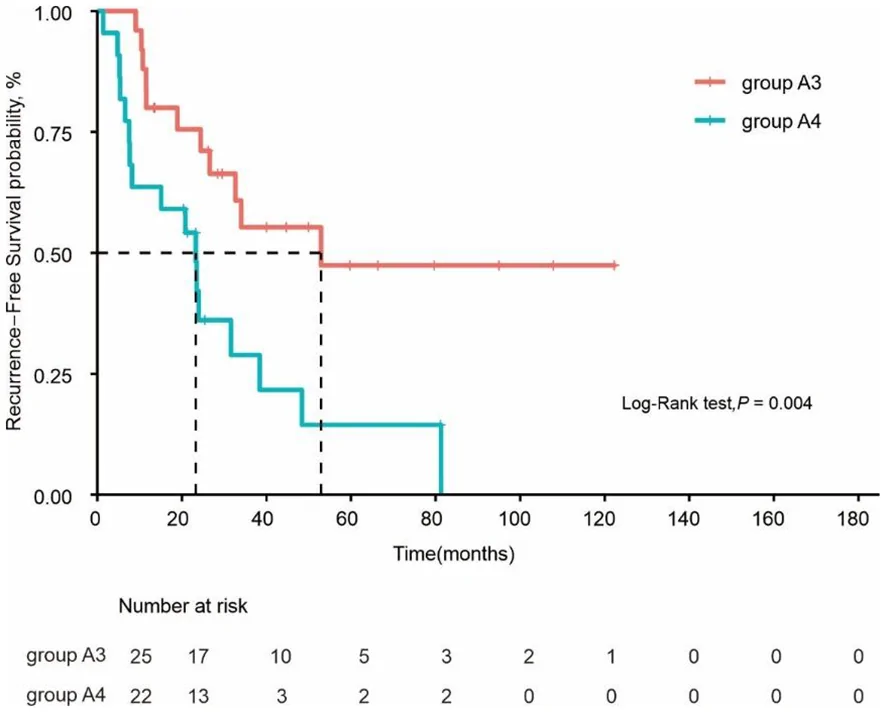
Kaplan-Meier analysis of RFS stratified by HD-IFN treatment duration in the high-risk group of AM patients. RFS, Relapse-Free Survival; HD-IFN, High-dose interferon-alpha-2b; AM, Acral Melanoma.

**Figure 14:**
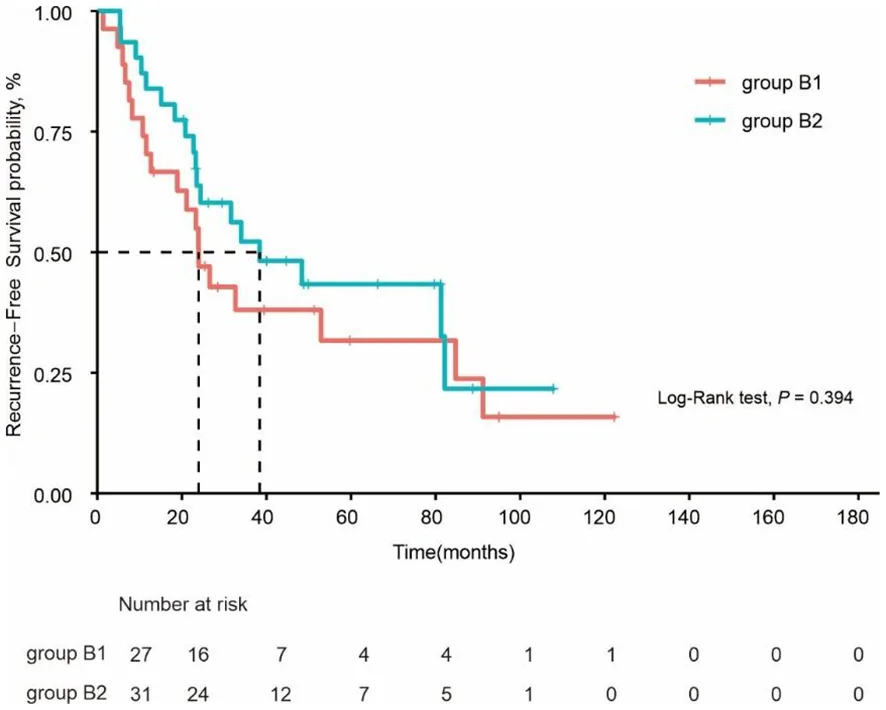
Kaplan-Meier analysis of RFS stratified by the interval from surgery to initial HD-IFN administration in the high-risk group of AM patients. RFS, Relapse-Free Survival; HD-IFN, High-dose interferon-alpha-2b; AM, Acral Melanoma.

The median RFS in groups A3 and A4 was 52.9 months and 23.3 months, respectively (*P* = 0.004) (**Figure 13**). The median RFS in Groups B1 and B2 were 23.9 months (95% CI 17.4–30.5) and 38.4 months (95% CI 14.1–62.7), respectively (*P* = 0.394) (**Figure 14**). Age, gender, presence or absence of ulcers, mitotic rate, and history of trauma were not associated with RFS.

In the univariate Cox proportional hazards model, the duration of IFN treatment was significantly associated with RFS in high-risk AM patients (**Supplementary Table 5**).

#### 3.2.6 Analysis of OS and prognostic factors in the high-risk group

**Figure 15:**
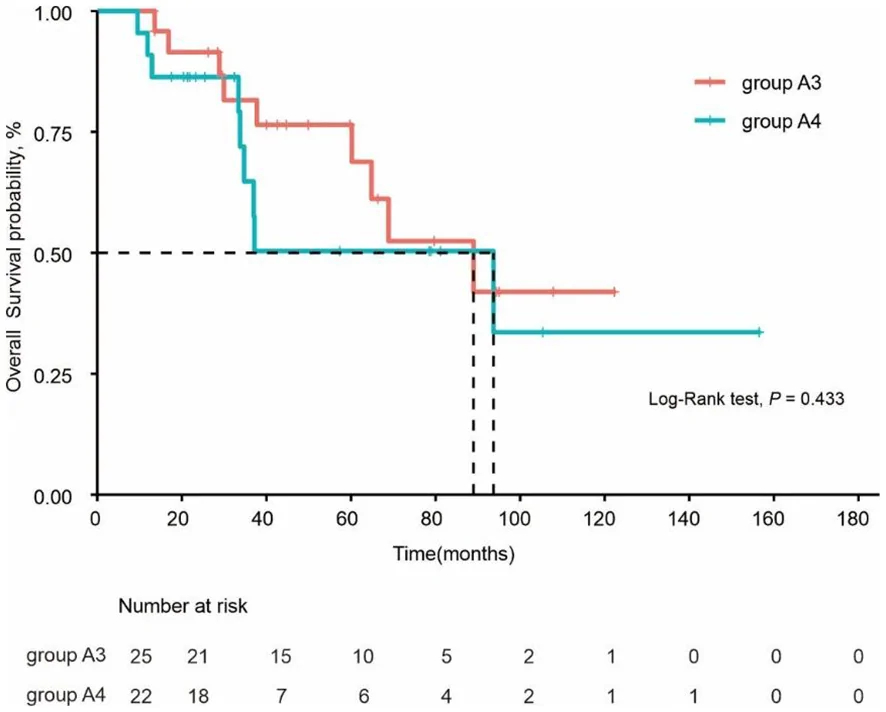
Kaplan-Meier analysis of OS stratified by HD-IFN treatment duration in the high-risk group of AM patients. OS, Overall Survival; HD-IFN, High-dose interferon-alpha-2b; AM, Acral Melanoma.

**Figure 16:**
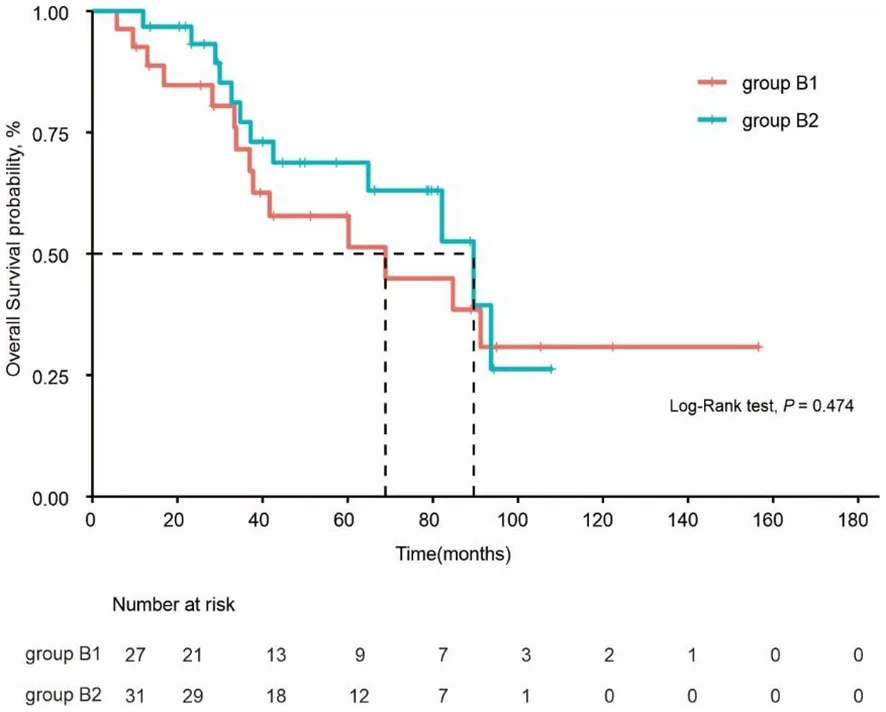
Kaplan-Meier analysis of OS stratified by the interval from surgery to initial HD-IFN administration in the high-risk group of AM patients. OS, Overall Survival; HD-IFN, High-dose interferon-alpha-2b; AM, Acral Melanoma.

The median OS for patients in groups A3 and A4 was 88.9 months (95% CI 54.2–123.7) and 93.7 months (95% CI 18.4–168.9), respectively (*P* = 0.433) (**Figure 15**). The median OS for patients in groups B1 and B2 were 68.9 months (95% CI 21.8–115.9) and 89.6 months (95% CI 59.1–120.1), respectively (*P* = 0.474) (**Figure 16**). Age, gender, presence or absence of ulcers, mitotic rate, and history of trauma were not significantly associated with OS.

Univariate analysis showed that none of the variables were significantly associated with OS, and no independent prognostic factors were identified (**Supplementary Table 6**).

#### 3.2.7 Analysis of RFS and prognostic factors in the very high-risk group

**Figure 17:**
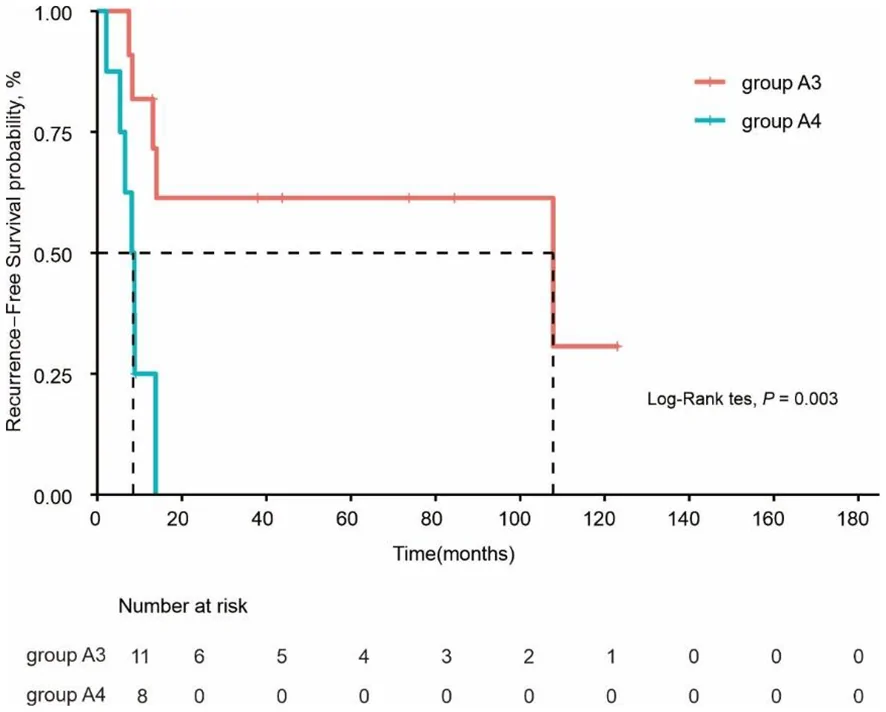
Kaplan-Meier analysis of RFS stratified by HD-IFN treatment duration in the very high-risk group of AM patients. RFS, Relapse-Free Survival; HD-IFN, High-dose interferon-alpha-2b; AM, Acral Melanoma.

**Figure 18:**
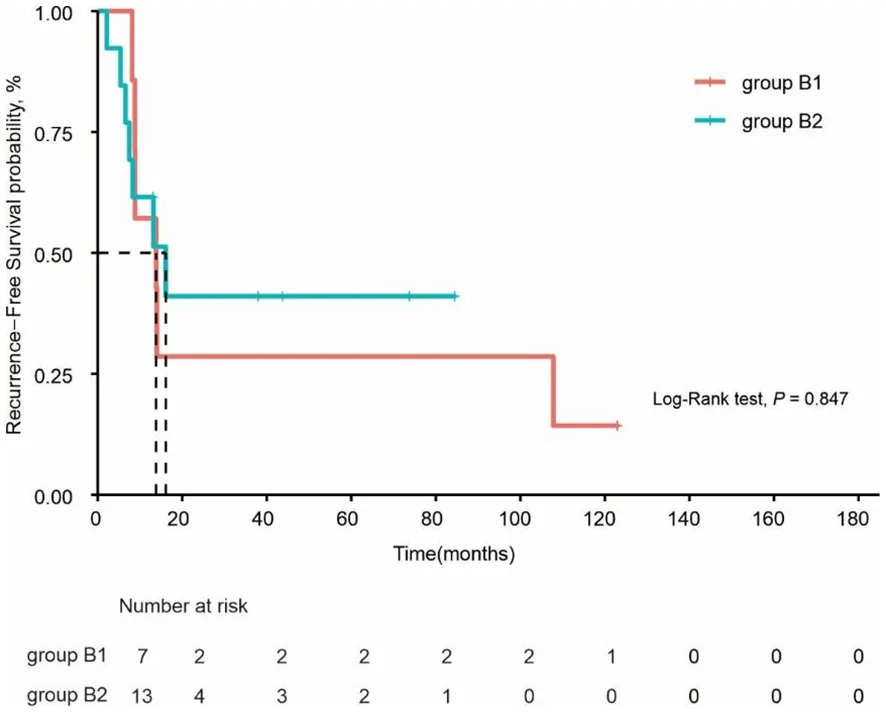
Kaplan-Meier analysis of RFS stratified by the interval from surgery to initial HD-IFN administration in the very high-risk group of AM patients. RFS, Relapse-Free Survival; HD-IFN, High-dose interferon-alpha-2b; AM, Acral Melanoma.

The median RFS was 107.8 months in Group A3 and 8.1 months in Group A4, with a statistically significant difference (*P* = 0.003) (**Figure 17**). In Groups B1 and B2, the median RFS was 13.8 months and 16.1 months, respectively (95% CI 4.9–27.2) (*P* = 0.847) (**Figure 18**). Age, gender, presence or absence of ulcers, mitotic rate, and history of trauma were not significantly associated with RFS.

In the univariate Cox proportional hazards model, the duration of IFN treatment was significantly associated with RFS in patients in the very high-risk group (**Supplementary Table 7**).

#### 3.2.8 Analysis of OS and prognostic factors in the very high-risk group

**Figure 19:**
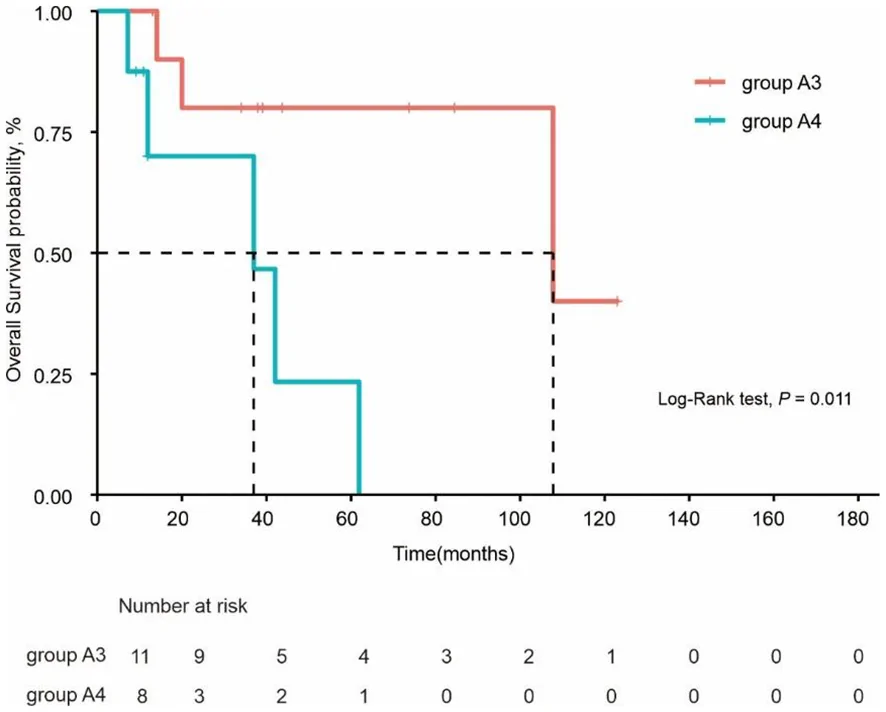
Kaplan-Meier analysis of OS stratified by HD-IFN treatment duration in the very high-risk group of AM patients. OS, Overall Survival; HD-IFN, High-dose interferon-alpha-2b; AM, Acral Melanoma.

**Figure 20:**
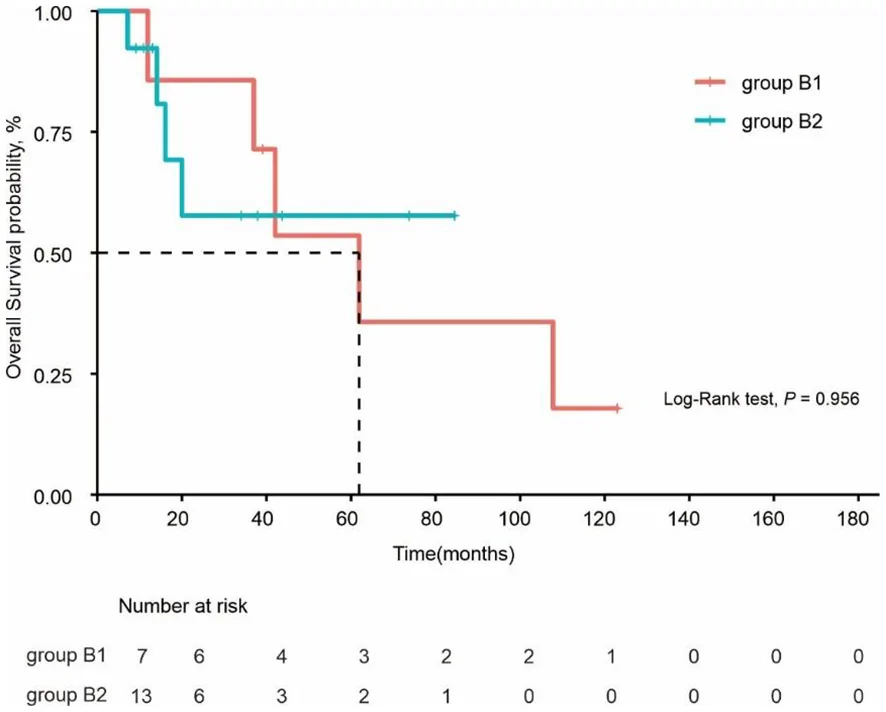
Kaplan-Meier analysis of OS stratified by the interval from surgery to initial HD-IFN administration in the very high-risk group of AM patients. OS, Overall Survival; HD-IFN, High-dose interferon-alpha-2b; AM, Acral Melanoma.

The median OS for patients in groups A3 and A4 was 107.8 months and 37.0 months, respectively (95% CI 8.4–65.6) (*P* = 0.011) (**Figure 19**). The 5-year OS rates for patients in Groups B1 and B2 were 53.6% and 57.7%, respectively (*P* = 0.956) (**Figure 20**). Patients younger than 60 years had a significantly longer median OS than those aged 60 years or older (*P* = 0.025). Gender, presence or absence of ulcers, mitotic rate, and history of trauma were not significantly associated with OS.

Univariate analysis showed that duration of IFN treatment and age were associated with OS in patients in the very high-risk group. Multivariate analysis did not identify any independent risk factors for OS in AM patients in the very high-risk group (**Supplementary Table 8**).

### 3.3 Safety and toxicity of HD-IFN

Among the 171 patients in this study, 2 had their dosage permanently reduced due to intolerance of HD-IFN-related adverse reactions, and 34 ultimately had their adjuvant therapy permanently discontinued due to the toxic side effects of HD-IFN. This study summarizes data on treatment completion during the induction and maintenance phases of HD-IFN therapy, reasons for discontinuation, and treatment-related adverse events, and compares differences in toxicity incidence among subgroups with different risk stratifications and treatment durations. Detailed statistical results are presented in **Supplementary Tables 9**–**15**.

## 4 Discussion

This real-world retrospective study includes the largest sample size to date evaluating HD-IFN adjuvant therapy for AM. We analyzed recurrence and survival outcomes in AM patients receiving postoperative HD-IFN therapy, examining outcomes by stage and risk stratification. Additionally, we explored the impact of HD-IFN treatment duration and the interval between surgery and the first dose on patient prognosis.

Previous retrospective studies have reported that the median RFS for patients with stage III AM receiving no adjuvant therapy was 11 months, compared to 15 months for those treated with HD-IFN (19). Another study showed that patients with stage III–IV AM without adjuvant therapy had median disease-free survival (DFS) and OS of 15.0 months and 38.0 months, respectively (20). In our study, HD-IFN adjuvant therapy in stage III AM resulted in a median RFS of 14.0 months and a median OS of 60.2 months. The median RFS and OS data for patients in this Phase III study show some numerical differences compared to the results from the historical cohort that had not previously received adjuvant therapy. Given that this study is a single-arm, retrospective cohort study without a concurrent parallel control group, comparisons with the literature can only serve as background information and a reference for research hypotheses. They can only suggest a potential trend toward a prognostic benefit with HD-IFN; they cannot directly confirm that HD-IFN definitively improves patients’ RFS and OS. The precise clinical efficacy of this regimen still requires validation through prospective controlled studies.

Numerous previous clinical trials have confirmed that HD-IFN can be used for adjuvant therapy following surgery for CM, however, the current mainstream first-line recommendation for adjuvant therapy for CM is a combination regimen of PD-1 inhibitors and BRAF/MEK targeted therapies. The E1694 trial reported a 24-month RFS rate of 62% in patients with stage IIB–III CM receiving HD-IFN (13). Similarly, the E1684 trial found a median RFS of 20.6 months for HD-IFN-treated stage IIB–III CM patients (14). European retrospective data showed 5-year DFS rates of 65.9% for polyethylene glycol interferon α-2b and 64.8% for low-dose interferon α-2b in stage IB–III melanoma (21). In comparison, our study found a 24-month RFS rate of 49.90% and median RFS of 23.9 months for HD-IFN-treated stage IIB–III AM, with 5-year RFS and OS rates of 43.50% and 70.30%, respectively. This study conducted a numerical comparison of the AM cohort data with the results of previous clinical trials of CM using interferon, and certain differences were observed in survival outcomes between the two groups. Due to limitations imposed by the study design and heterogeneity in baseline data, this comparison is provided solely as background information and does not directly compare the efficacy of interferon in the two subtypes.

A study by Chinese researchers reported median RFS times of 17.9 and 22.5 months for stage IIB–IIIC AM patients treated with 1-month and 1-year HD-IFN regimens, respectively (18). The median RFS observed in this study was 23.9 months, which is comparable to the results of previous retrospective cohort studies in China using the same treatment regimen. These findings provide real-world clinical data on the use of HD-IFN as adjuvant therapy for AM and can serve as a reference for the design of future prospective controlled studies.

Single-arm retrospective studies investigating adjuvant PD-1 inhibitor therapy for AM fail to provide high-level evidence-based medical evidence. One study with 31 patients with stage IIIA–IIID AM reported a 12-month RFS rate of only 23.00% (22). Another study with 44 stage II–III AM patients treated with PD-1 inhibitors showed a median RFS of 21.6 months (23). By comparison, our study found a 12-month RFS rate of 60.0% in stage IIIB–IIID patients and a median RFS of 31.6 months in stage II–III patients receiving HD-IFN. A literature review comparing the data from this cohort with the results of two single-arm retrospective studies on adjuvant therapy with PD-1 monoclonal antibodies revealed differences in survival outcomes between the two cohorts. Due to differences in study design and baseline characteristics, it is not possible to confirm that HD-IFN is more effective; this requires verification through future randomized controlled trials.

In the low-risk group, patients in group A3 achieved better RFS compared to groups A1 and A2. Although group A1 also showed better RFS than group A2, the smaller sample size in group A2 may have introduced bias. Among high-risk patients, group A3 had significantly longer RFS than group A4, but no significant difference in OS was found between different treatment durations in low- and high-risk groups. Compared with Group A4, Group A3 patients classified as very high-risk showed statistically significant differences in RFS and OS, with longer survival times. The results of this study are consistent with trends observed in previous studies, showing differences in survival outcomes among different treatment groups in high-risk populations (18). Subgroup analysis results show that, in high-risk and very high-risk populations, a full-course of HD-IFN therapy is statistically associated with improved OS; however, given the limitations of the retrospective study design and the relatively small sample size in the very high-risk subgroup, these findings may serve as a reference for clinicians when determining the duration of individualized adjuvant therapy.

In this cohort, initiating HD-IFN treatment at different time points within 3 months postoperatively showed no significant association with patient recurrence or survival outcomes, and this trend remained consistent across the overall population and all risk strata. Analysis of the overall population indicated that age and the presence of an ulcer at the primary site are prognostic factors associated with RFS and OS in patients with AM; patients aged 60 years or older and those with an ulcer had a poorer prognosis, consistent with the findings of previous studies (24, 25). Stratified analysis revealed differences in the prognostic effects of these two factors across subgroups: within the low-risk and high-risk groups, baseline factors such as age and ulceration showed no statistically significant association with RFS or OS; in the very high-risk group, only age was associated with OS, with younger patients exhibiting better OS, while age had no significant impact on RFS in this group.

This study has several inherent limitations: First, the retrospective cohort study is subject to selection bias, as only patients who were able to tolerate and initiated HD-IFN therapy were included among the 845 consecutive cases. Cases involving early disease progression, intolerance to toxicity, or the use of other adjuvant regimens were excluded, which may have led to an overly optimistic survival outcome; therefore, the conclusions cannot be generalized to all patients with AM. Second, the sample sizes across the various risk subgroups were uneven, with a relatively small number of cases in the very high-risk group, limiting the statistical power of the subgroup analysis; third, the study included only a single-center cohort in China, limiting the generalizability of the findings to other populations. Fourth, classifying treatment courses based on the final duration of medication use during follow-up introduces a persistent time bias. Due to sample size limitations, it was not possible to perform adjustments using landmark analysis or time-dependent Cox regression; therefore, only observational results are presented. Further prospective studies are needed to validate these conclusions. Fifth, the X-tile method was used to determine the treatment cutoff value within this dataset; however, there was no validation using an external cohort, and no correction for multiple comparisons was performed. This stratification was conducted solely for exploratory analysis, and the findings of this study require validation through large-scale prospective studies. Furthermore, due to limitations in the completeness of the retrospective medical records, there was a high rate of missing data for the Clark classification, Ki-67 index, microsatellite status, and sentinel lymph node biopsy results, which may introduce some information bias and make it difficult to conduct in-depth subgroup analyses based on these indicators.

## 5 Conclusion

This single-arm, retrospective cohort study describes recurrence and survival outcomes in patients with AM who received 1 year of HD-IFN adjuvant therapy postoperatively. In the overall cohort, age and primary lesion ulceration were baseline prognostic factors. Stratified analysis indicated that, in the high-risk and very high-risk subgroups, a full course of IFN intervention was statistically associated with better RFS and OS. However, no significant impact on patient prognosis was observed based on different intervals for initiating treatment within the first 3 months postoperatively. This study merely provides supplementary real-world observational data from the Chinese population and cannot confirm that HD-IFN offers clear clinical benefits or therapeutic advantages. Future randomized controlled trials are needed to further validate the clinical value of HD-IFN, thereby enabling the development of individualized adjuvant treatment strategies based on risk stratification.

## Data Availability

The raw data supporting the conclusions of this article will be made available by the authors, without undue reservation.

## Supplementary material

Supplementary Figure 1 CONSORT flow diagram of participant screening and enrollment.

Supplementary Table 1 Univariate and multivariate Cox regression analyses for RFS in the total population. HD-IFN, High-Dose Interferon α-2b; RFS, Relapse-Free Survival; P, P-value.

Supplementary Table 2 Univariate and multivariate Cox regression analyses for OS in the total population. HD-IFN, High-Dose Interferon α-2b; OS, Overall Survival; P, P-value.

Supplementary Table 3 Univariate and multivariate Cox regression analyses for RFS in the low-risk group. HD-IFN, High-Dose Interferon α-2b; RFS, Relapse-Free Survival; P, P-value.

Supplementary Table 4 Univariate and multivariate Cox regression analyses for OS in the low-risk population. HD-IFN, High-Dose Interferon α-2b; OS, Overall Survival; P, P-value.

Supplementary Table 5 Univariate and multivariate Cox regression analyses for RFS in the high-risk group. HD-IFN, High-Dose Interferon α-2b; RFS, Relapse-Free Survival; P, P-value.

Supplementary Table 6 Univariate and multivariate Cox regression analyses for OS in the high-risk population. HD-IFN, High-Dose Interferon α-2b; OS, Overall Survival; P, P-value.

Supplementary Table 7 Univariate and multivariate Cox regression analyses for RFS in the very high-risk group. HD-IFN, High-Dose Interferon α-2b; RFS, Relapse-Free Survival; P, P-value.

Supplementary Table 8 Univariate and multivariate Cox regression analyses for OS in the very high-risk population. HD-IFN, High-Dose Interferon α-2b; OS, Overall Survival; P, P-value.

Supplementary Table 9 Completion status of HD-IFN treatment duration.

Supplementary Table 10 Composition of treatment discontinuation reasons.

Supplementary Table 11 Overview of treatment-related adverse events.

Supplementary Table 12 Comparison of the incidence of any adverse events across different risk subgroups.

Supplementary Table 13 Comparison of the incidence of grade ≥3 adverse events among different risk subgroups.

Supplementary Table 14 Comparison of the incidence of any adverse events among subgroups with different treatment durations.

Supplementary Table 15 Comparison of the incidence of grade ≥3 adverse events among subgroups with different treatment durations.
