# Supplementary material for: High dose IFN-α2b as post operative adjuvant therapy for acral melanoma: a multi-center real-world cohort study of 171 Chinese patients
Source: Front Oncol. 2026 Aug 17;16:1886308. doi: 10.3389/fonc.2026.1886308 (PMC13526564; doi:10.3389/fonc.2026.1886308)
Supplement: Supplementary file 1 [file SupplementaryFile1.zip › Supplementary Tables and figure/Supplementary Table 1.docx]

| **Factors** | **Univariate Analysis** | | |  | **Multivariate Analysis** | | |
| --- | --- | --- | --- | --- | --- | --- | --- |
|  | **HR** | **95%CI** | **P** |  | **HR** | **95%CI** | **P** |
| **Age, y** |  |  | **0.008** |  |  |  | **0.116** |
| ＜60 | 1 | Reference |  |  | 1 | Reference |  |
| ≥60 | 1.75 | 1.16-2.65 | 0.008 |  | 1.54 | 0.41-1.10 | 0.116 |
| **Gender** |  |  | **0.126** |  |  |  |  |
| Male | 1 | Reference |  |  |  |  |  |
| Female | 0.73 | 0.48-1.09 | 0.126 |  |  |  |  |
| **Ulceration** |  |  | **0.007** |  |  |  | **0.065** |
| Yes | 1 | Reference |  |  | 1 | Reference |  |
| No | 0.54 | 0.34-0.84 | 0.007 |  | 0.59 | 0.97-2.68 | 0.065 |
| **Mitotic rate/mm^2^** |  |  | **0.482** |  |  |  |  |
| ＜1 | 1 | Reference |  |  |  |  |  |
| ≥1 | 1.35 | 0.58-3.14 | 0.482 |  |  |  |  |
| **Trauma** |  |  | **0.621** |  |  |  |  |
| Yes | 1 | Reference |  |  |  |  |  |
| No | 0.89 | 0.58-1.38 | 0.621 |  |  |  |  |
| **Duration of HD-IFN treatment** |  |  | **＜0.001** |  |  |  | **＜0.001** |
| Group A1 | 1 | Reference |  |  | 1 | Reference |  |
| Group A2 | 2.75 | 1.53-4.95 | ＜0.001 |  | 2.68 | 1.48-4.88 | 0.001 |
| Group A3 | 0.76 | 0.43-1.36 | 0.366 |  | 0.72 | 0.39-1.31 | 0.287 |
| **Surgery-to-first-IFN interval** |  |  | **0.421** |  |  |  |  |
| Group B1 | 1 | Reference |  |  |  |  |  |
| Group B2 | 0.84 | 0.56-1.28 | 0.421 |  |  |  |  |

**Supplementary Table 1. Univariate and multivariate Cox regression analyses for RFS in the total population.**

HD-IFN：High-Dose Interferon α-2b

RFS：Relapse-Free Survival

P: P-value
