# Supplementary material for: High dose IFN-α2b as post operative adjuvant therapy for acral melanoma: a multi-center real-world cohort study of 171 Chinese patients
Source: Front Oncol. 2026 Aug 17;16:1886308. doi: 10.3389/fonc.2026.1886308 (PMC13526564; doi:10.3389/fonc.2026.1886308)
Supplement: Supplementary file 1 [file SupplementaryFile1.zip › Supplementary Tables and figure/Supplementary Table 10.docx]

| **Variable** | **No. of patients (%)**  **（n=49）** |
| --- | --- |
| **Intolerance to interferon-associated adverse events** | 34（69.4） |
| **Recurrence/progression during follow-up** | 3（6.1） |
| **Poor adherence** | 3（6.1） |
| **Exacerbated comorbidities plus vital organ dysfunction** | 4（8.2） |
| **Other clinical causes** | 5（10.2） |

**Supplementary Table 10.** **Composition of treatment discontinuation reasons.**
