# Supplementary material for: High dose IFN-α2b as post operative adjuvant therapy for acral melanoma: a multi-center real-world cohort study of 171 Chinese patients
Source: Front Oncol. 2026 Aug 17;16:1886308. doi: 10.3389/fonc.2026.1886308 (PMC13526564; doi:10.3389/fonc.2026.1886308)
Supplement: Supplementary file 1 [file SupplementaryFile1.zip › Supplementary Tables and figure/Supplementary Table 11.docx]

| **Category of Adverse Events** | **Cases** | **Overall Incidence** | **Number of Grade 3–4 Toxicities** | **Grade 3–4 Toxicity Incidence** |
| --- | --- | --- | --- | --- |
| **Influenza-like symptoms** | 53 | 31.0% | 8 | 4.7% |
| **Myelosuppression** | 61 | 35.7% | 6 | 3.5% |
| **Elevated liver transaminases** | 31 | 18.1% | 9 | 5.3% |
| **Interferon-related retinopathy** | 6 | 3.5% | 0 | 0.0% |
| **Gastrointestinal adverse reactions** | 19 | 11.1% | 6 | 3.5% |
| **Psychoneurological symptoms** | 3 | 1.8% | 1 | 0.6% |
| **Other adverse reactions** | 5 | 2.9% | 2 | 1.2% |

**Supplementary Table 11.** **Overview of treatment-related adverse events.**
