# Supplementary material for: High dose IFN-α2b as post operative adjuvant therapy for acral melanoma: a multi-center real-world cohort study of 171 Chinese patients
Source: Front Oncol. 2026 Aug 17;16:1886308. doi: 10.3389/fonc.2026.1886308 (PMC13526564; doi:10.3389/fonc.2026.1886308)
Supplement: Supplementary file 1 [file SupplementaryFile1.zip › Supplementary Tables and figure/Supplementary Table 13.docx]

| **Risk Stratification** | **Total (n)** | **Incidence of Any Adverse Event [n (%)]** | **Overall P-value** | **Intergroup Comparison Notes** |
| --- | --- | --- | --- | --- |
| **Low-risk group** | 63 | 5（7.9） | 0.578 |  |
| **High-risk group** | 63 | 48（11.1） |  |  |
| **Very-high-risk group** | 20 | 14（5.0） |  |  |

**Supplementary Table 13.** **Comparison of the incidence of grade ≥3 adverse events among different risk subgroups.**
