# Supplementary material for: High dose IFN-α2b as post operative adjuvant therapy for acral melanoma: a multi-center real-world cohort study of 171 Chinese patients
Source: Front Oncol. 2026 Aug 17;16:1886308. doi: 10.3389/fonc.2026.1886308 (PMC13526564; doi:10.3389/fonc.2026.1886308)
Supplement: Supplementary file 1 [file SupplementaryFile1.zip › Supplementary Tables and figure/Supplementary Table 15.docx]

| **Variable** | **Total (n)** | **Incidence of Any Adverse Event [n (%)]** | **Overall P-value** | **Intergroup Comparison Notes** |
| --- | --- | --- | --- | --- |
| **Completed induction-phase therapy** | 59 | 9（15.3） | 0.043 | 0.014 |
| **Completed full-course induction and maintenance regimen** | 49 | 1（2.0） |  | Reference |
| **Intensive phase completed; maintenance phase unfinished** | 24 | 1（4.2） |  | No significant pairwise differences |

**Supplementary Table 15.** **Comparison of the incidence of grade ≥3 adverse events among subgroups with different treatment durations.**
