# Supplementary material for: High dose IFN-α2b as post operative adjuvant therapy for acral melanoma: a multi-center real-world cohort study of 171 Chinese patients
Source: Front Oncol. 2026 Aug 17;16:1886308. doi: 10.3389/fonc.2026.1886308 (PMC13526564; doi:10.3389/fonc.2026.1886308)
Supplement: Supplementary file 1 [file SupplementaryFile1.zip › Supplementary Tables and figure/Supplementary Table 2.docx]

| **Factors** | **Univariate Analysis** | | |  | **Multivariate Analysis** | | |
| --- | --- | --- | --- | --- | --- | --- | --- |
|  | **HR** | **95%CI** | **P** |  | **HR** | **95%CI** | **P** |
| **Age, y** |  |  | **0.034** |  |  |  | **0.102** |
| ＜60 | 1 | Reference |  |  | 1 | Reference |  |
| ≥60 | 1.73 | 1.04-2.86 | 0.034 |  | 1.78 | 0.31-1.11 | 0.102 |
| **Gender** |  |  | **0.079** |  |  |  |  |
| Male | 1 | Reference |  |  |  |  |  |
| Female | 0.64 | 0.39-1.05 | 0.079 |  |  |  |  |
| **Ulceration** |  |  | **0.009** |  |  |  | **0.167** |
| Yes | 1 | Reference |  |  | 1 | Reference |  |
| No | 0.47 | 0.27-0.83 | 0.009 |  | 0.59 | 0.31-1.11 | 0.167 |
| **Mitotic rate/mm^2^** |  |  | **0.383** |  |  |  |  |
| ＜1 | 1 | Reference |  |  |  |  |  |
| ≥1 | 1.69 | 0.52-5.48 | 0.383 |  |  |  |  |
| **Trauma** |  |  | **0.743** |  |  |  |  |
| Yes | 1 | Reference |  |  |  |  |  |
| No | 1.09 | 0.64-1.88 | 0.743 |  |  |  |  |
| **Duration of HD-IFN treatment** |  |  | **0.483** |  |  |  |  |
| Group A1 | 1 | Reference |  |  |  |  |  |
| Group A2 | 1.13 | 0.51-2.51 | 0.759 |  |  |  |  |
| Group A3 | 0.72 | 0.34-1.45 | 0.357 |  |  |  |  |
| **Surgery-to-first-IFN interval** |  |  | **0.932** |  |  |  |  |
| Group B1 | 1 | Reference |  |  |  |  |  |
| Group B2 | 0.98 | 0.59-1.63 | 0.932 |  |  |  |  |

**Supplementary Table 2. Univariate and multivariate Cox regression analyses for OS in the total population.**

HD-IFN：High-Dose Interferon α-2b

OS：Overall Survival

P: P-value
