# Supplementary material for: High dose IFN-α2b as post operative adjuvant therapy for acral melanoma: a multi-center real-world cohort study of 171 Chinese patients
Source: Front Oncol. 2026 Aug 17;16:1886308. doi: 10.3389/fonc.2026.1886308 (PMC13526564; doi:10.3389/fonc.2026.1886308)
Supplement: Supplementary file 1 [file SupplementaryFile1.zip › Supplementary Tables and figure/Supplementary Table 3.docx]

| **Factors** | **Univariate Analysis** | | |  | **Multivariate Analysis** | | |
| --- | --- | --- | --- | --- | --- | --- | --- |
|  | **HR** | **95%CI** | **P** |  | **HR** | **95%CI** | **P** |
| **Age, y** |  |  | **0.065** |  |  |  |  |
| ＜60 | 1 | Reference |  |  |  |  |  |
| ≥60 | 2.09 | 0.96-4.56 | 0.065 |  |  |  |  |
| **Gender** |  |  | **0.927** |  |  |  |  |
| Male | 1 | Reference |  |  |  |  |  |
| Female | 1.04 | 0.46-2.34 | 0.927 |  |  |  |  |
| **Ulceration** |  |  | **0.372** |  |  |  |  |
| Yes | 1 | Reference |  |  |  |  |  |
| No | 0.69 | 0.1-1.55 | 0.372 |  |  |  |  |
| **Mitotic rate/mm^2^** |  |  | **0.945** |  |  |  |  |
| ＜1 | 1 | Reference |  |  |  |  |  |
| ≥1 | 0.96 | 0.33-2.84 | 0.945 |  |  |  |  |
| **Trauma** |  |  | **0.196** |  |  |  |  |
| Yes | 1 | Reference |  |  |  |  |  |
| No | 0.59 | 0.27-1.31 | 0.196 |  |  |  |  |
| **Duration of HD-IFN treatment** |  |  | **0.015** |  |  |  |  |
| Group A1 | 1 | Reference |  |  |  |  |  |
| Group A2 | 3.62 | 1.37-9.52 | 0.009 |  |  |  |  |
| Group A3 | 0.83 | 0.26-2.66 | 0.758 |  |  |  |  |
| **Surgery-to-first-IFN interval** |  |  | **0.818** |  |  |  |  |
| Group B1 | 1 | Reference |  |  |  |  |  |
| Group B2 | 0.92 | 0.44-1.91 | 0.818 |  |  |  |  |

**Supplementary Table 3. Univariate and multivariate Cox regression analyses for RFS in the low-risk group.**

HD-IFN：High-Dose Interferon α-2b

RFS：Relapse-Free Survival

P: P-value
