# Supplementary material for: High dose IFN-α2b as post operative adjuvant therapy for acral melanoma: a multi-center real-world cohort study of 171 Chinese patients
Source: Front Oncol. 2026 Aug 17;16:1886308. doi: 10.3389/fonc.2026.1886308 (PMC13526564; doi:10.3389/fonc.2026.1886308)
Supplement: Supplementary file 1 [file SupplementaryFile1.zip › Supplementary Tables and figure/Supplementary Table 4.docx]

| **Factors** | **Univariate Analysis** | | |  | **Multivariate Analysis** | | |
| --- | --- | --- | --- | --- | --- | --- | --- |
|  | **HR** | **95%CI** | **P** |  | **HR** | **95%CI** | **P** |
| **Age, y** |  |  | **0.087** |  |  |  |  |
| ＜60 | 1 | Reference |  |  |  |  |  |
| ≥60 | 2.47 | 0.88-6.94 | 0.087 |  |  |  |  |
| **Gender** |  |  | **0.703** |  |  |  |  |
| Male | 1 | Reference |  |  |  |  |  |
| Female | 1.23 | 0.420-3.62 | 0.703 |  |  |  |  |
| **Ulceration** |  |  | **0.390** |  |  |  |  |
| Yes | 1 | Reference |  |  |  |  |  |
| No | 0.65 | 0.24-1.75 | 0.390 |  |  |  |  |
| **Mitotic rate/mm^2^** |  |  | **0.799** |  |  |  |  |
| ＜1 | 1 | Reference |  |  |  |  |  |
| ≥1 | 1.21 | 0.27-5.42 | 0.799 |  |  |  |  |
| **Trauma** |  |  | **0.220** |  |  |  |  |
| Yes | 1 | Reference |  |  |  |  |  |
| No | 0.53 | 0.19-1.47 | 0.220 |  |  |  |  |
| **Duration of HD-IFN treatment** |  |  | **0.477** |  |  |  |  |
| Group A1 | 1 | Reference |  |  |  |  |  |
| Group A2 | 1.45 | 0.38-5.48 | 0.585 |  |  |  |  |
| Group A3 | 0.57 | 0.13-2.51 | 0.456 |  |  |  |  |
| **Surgery-to-first-IFN interval** |  |  | **0.496** |  |  |  |  |
| Group B1 | 1 | Reference |  |  |  |  |  |
| Group B2 | 1.42 | 0.52-3.86 | 0.496 |  |  |  |  |

**Supplementary Table 4. Univariate and multivariate Cox regression analyses for OS in the low-risk population.**

HD-IFN：High-Dose Interferon α-2b

OS：Overall Survival

P: P-value
