# Supplementary material for: High dose IFN-α2b as post operative adjuvant therapy for acral melanoma: a multi-center real-world cohort study of 171 Chinese patients
Source: Front Oncol. 2026 Aug 17;16:1886308. doi: 10.3389/fonc.2026.1886308 (PMC13526564; doi:10.3389/fonc.2026.1886308)
Supplement: Supplementary file 1 [file SupplementaryFile1.zip › Supplementary Tables and figure/Supplementary Table 5.docx]

| **Factors** | **Univariate Analysis** | | |  | **Multivariate Analysis** | | |
| --- | --- | --- | --- | --- | --- | --- | --- |
|  | **HR** | **95%CI** | **P** |  | **HR** | **95%CI** | **P** |
| **Age, y** |  |  | **0.373** |  |  |  |  |
| ＜60 | 1 | Reference |  |  |  |  |  |
| ≥60 | 1.33 | 0.71-2.47 | 0.373 |  |  |  |  |
| **Gender** |  |  | **0.083** |  |  |  |  |
| Male | 1 | Reference |  |  |  |  |  |
| Female | 0.58 | 0.31-1.08 | 0.083 |  |  |  |  |
| **Ulceration** |  |  | **0.095** |  |  |  |  |
| Yes | 1 | Reference |  |  |  |  |  |
| No | 0.47 | 0.19-1.14 | 0.095 |  |  |  |  |
| **Mitotic rate/mm^2^** |  |  | **0.645** |  |  |  |  |
| ＜1 | 1 | Reference |  |  |  |  |  |
| ≥1 | 1.60 | 0.22-11.96 | 0.645 |  |  |  |  |
| **Trauma** |  |  | **0.774** |  |  |  |  |
| Yes | 1 | Reference |  |  |  |  |  |
| No | 1.10 | 0.57-2.12 | 0.774 |  |  |  |  |
| **Duration of HD-IFN treatment** |  |  | **0.006** |  |  |  |  |
| Group A3 | 1 | Reference |  |  |  |  |  |
| Group A4 | 2.92 | 1.35-6.30 | 0.006 |  |  |  |  |
| **Surgery-to-first-IFN interval** |  |  | **0.395** |  |  |  |  |
| Group B1 | 1 | Reference |  |  |  |  |  |
| Group B2 | 0.75 | 0.39-1.45 | 0.395 |  |  |  |  |

**Supplementary Table 5. Univariate and multivariate Cox regression analyses for RFS in the high-risk group.**

HD-IFN：High-Dose Interferon α-2b

RFS：Relapse-Free Survival

P: P-value
