# Supplementary material for: High dose IFN-α2b as post operative adjuvant therapy for acral melanoma: a multi-center real-world cohort study of 171 Chinese patients
Source: Front Oncol. 2026 Aug 17;16:1886308. doi: 10.3389/fonc.2026.1886308 (PMC13526564; doi:10.3389/fonc.2026.1886308)
Supplement: Supplementary file 1 [file SupplementaryFile1.zip › Supplementary Tables and figure/Supplementary Table 6.docx]

| **Factors** | **Univariate Analysis** | | |  | **Multivariate Analysis** | | |
| --- | --- | --- | --- | --- | --- | --- | --- |
|  | **HR** | **95%CI** | **P** |  | **HR** | **95%CI** | **P** |
| **Age, y** |  |  | **0.870** |  |  |  |  |
| ＜60 | 1 | Reference |  |  |  |  |  |
| ≥60 | 0.94 | 0.44-2.01 | 0.870 |  |  |  |  |
| **Gender** |  |  | **0.985** |  |  |  |  |
| Male | 1 | Reference |  |  |  |  |  |
| Female | 0.99 | 0.47-2.09 | 0.985 |  |  |  |  |
| **Ulceration** |  |  | **0.160** |  |  |  |  |
| Yes | 1 | Reference |  |  |  |  |  |
| No | 0.47 | 0.16-1.35 | 0.160 |  |  |  |  |
| **Mitotic rate/mm^2^** |  |  | **0.818** |  |  |  |  |
| ＜1 | 1 | Reference |  |  |  |  |  |
| ≥1 | 1.27 | 0.17-9.65 | 0.818 |  |  |  |  |
| **Trauma** |  |  | **0.360** |  |  |  |  |
| Yes | 1 | Reference |  |  |  |  |  |
| No | 1.48 | 0.64-3.39 | 0.360 |  |  |  |  |
| **Duration of HD-IFN treatment** |  |  | **0.436** |  |  |  |  |
| Group A3 | 1 | Reference |  |  |  |  |  |
| Group A4 | 1.45 | 0.57-3.66 | 0.436 |  |  |  |  |
| **Surgery-to-first-IFN interval** |  |  | **0.476** |  |  |  |  |
| Group B1 | 1 | Reference |  |  |  |  |  |
| Group B2 | 0.75 | 0.35-1.64 | 0.476 |  |  |  |  |

**Supplementary Table 6. Univariate and multivariate Cox regression analyses for OS in the high-risk population.**

HD-IFN：High-Dose Interferon α-2b

OS：Overall Survival

P: P-value
