# Supplementary material for: High dose IFN-α2b as post operative adjuvant therapy for acral melanoma: a multi-center real-world cohort study of 171 Chinese patients
Source: Front Oncol. 2026 Aug 17;16:1886308. doi: 10.3389/fonc.2026.1886308 (PMC13526564; doi:10.3389/fonc.2026.1886308)
Supplement: Supplementary file 1 [file SupplementaryFile1.zip › Supplementary Tables and figure/Supplementary Table 7.docx]

| **Factors** | **Univariate Analysis** | | |  | **Multivariate Analysis** | | |
| --- | --- | --- | --- | --- | --- | --- | --- |
|  | **HR** | **95%CI** | **P** |  | **HR** | **95%CI** | **P** |
| **Age, y** |  |  | **0.201** |  |  |  |  |
| ＜60 | 1 | Reference |  |  |  |  |  |
| ≥60 | 2.23 | 0.65-7.63 | 0.201 |  |  |  |  |
| **Gender** |  |  | **0.405** |  |  |  |  |
| Male | 1 | Reference |  |  |  |  |  |
| Female | 0.59 | 0.18-2.02 | 0.405 |  |  |  |  |
| **Ulceration** |  |  | **0.866** |  |  |  |  |
| Yes | 1 | Reference |  |  |  |  |  |
| No | 1.11 | 0.33-3.69 | 0.866 |  |  |  |  |
| **Mitotic rate/mm^2^** |  |  | **0.224** |  |  |  |  |
| ＜1 | 1 | Reference |  |  |  |  |  |
| ≥1 | 3.63 | 0.45-28.93 | 0.224 |  |  |  |  |
| **Trauma** |  |  | **0.980** |  |  |  |  |
| Yes | 1 | Reference |  |  |  |  |  |
| No | 0.98 | 0.31-3.15 | 0.980 |  |  |  |  |
| **Duration of HD-IFN treatment** |  |  | **0.009** |  |  |  |  |
| Group A3 | 1 | Reference |  |  |  |  |  |
| Group A4 | 6.49 | 1.59-26.46 | 0.009 |  |  |  |  |
| **Surgery-to-first-IFN interval** |  |  | **0.847** |  |  |  |  |
| Group B1 | 1 | Reference |  |  |  |  |  |
| Group B2 | 0.89 | 0.28-2.83 | 0.847 |  |  |  |  |

**Supplementary Table 7. Univariate and multivariate Cox regression analyses for RFS in the very high-risk group.**

HD-IFN：High-Dose Interferon α-2b

RFS：Relapse-Free Survival

P: P-value
