# Supplementary material for: High dose IFN-α2b as post operative adjuvant therapy for acral melanoma: a multi-center real-world cohort study of 171 Chinese patients
Source: Front Oncol. 2026 Aug 17;16:1886308. doi: 10.3389/fonc.2026.1886308 (PMC13526564; doi:10.3389/fonc.2026.1886308)
Supplement: Supplementary file 1 [file SupplementaryFile1.zip › Supplementary Tables and figure/Supplementary Table 8.docx]

| **Factors** | **Univariate Analysis** | | |  | **Multivariate Analysis** | | |
| --- | --- | --- | --- | --- | --- | --- | --- |
|  | **HR** | **95%CI** | **P** |  | **HR** | **95%CI** | **P** |
| **Age, y** |  |  | **0.042** |  |  |  | **0.134** |
| ＜60 | 1 | Reference |  |  | 1 | Reference |  |
| ≥60 | 5.42 | 1.06-27.71 | 0.042 |  | 5.92 | 0.58-60.53 | 0.134 |
| **Gender** |  |  | **0.531** |  |  |  |  |
| Male | 1 | Reference |  |  |  |  |  |
| Female | 0.59 | 0.12-2.98 | 0.531 |  |  |  |  |
| **Ulceration** |  |  | **0.531** |  |  |  |  |
| Yes | 1 | Reference |  |  |  |  |  |
| No | 0.59 | 0.12-2.98 | 0.531 |  |  |  |  |
| **Mitotic rate/mm^2^** |  |  | **0.301** |  |  |  |  |
| ＜1 | 1 | Reference |  |  |  |  |  |
| ≥1 | 36.38 | 0.04-328.75 | 0.301 |  |  |  |  |
| **Trauma** |  |  | **0.335** |  |  |  |  |
| Yes | 1 | Reference |  |  |  |  |  |
| No | 0.52 | 0.14-1.96 | 0.335 |  |  |  |  |
| **Duration of HD-IFN treatment** |  |  | **0.026** |  |  |  | **0.243** |
| Group A3 | 1 | Reference |  |  | 1 | Reference |  |
| Group A4 | 6.52 | 1.25-34.15 | 0.026 |  | 3.01 | 0.47-19.06 | 0.243 |
| **Surgery-to-first-IFN interval** |  |  | **0.956** |  |  |  |  |
| Group B1 | 1 | Reference |  |  |  |  |  |
| Group B2 | 0.96 | 0.24-3.93 | 0.956 |  |  |  |  |

**Supplementary Table 8. Univariate and multivariate Cox regression analyses for OS in the very high-risk population.**

HD-IFN：High-Dose Interferon α-2b

OS：Overall Survival

P: P-value
