# Supplementary material for: High dose IFN-α2b as post operative adjuvant therapy for acral melanoma: a multi-center real-world cohort study of 171 Chinese patients
Source: Front Oncol. 2026 Aug 17;16:1886308. doi: 10.3389/fonc.2026.1886308 (PMC13526564; doi:10.3389/fonc.2026.1886308)
Supplement: Supplementary file 1 [file SupplementaryFile1.zip › Supplementary Tables and figure/Supplementary Table 9.docx]

| **Variable** | **No. of patients (%)**  **（n=171）** |
| --- | --- |
| **Completed induction-phase therapy** | 59（34.5） |
| **Completed full-course induction and maintenance regimen** | 49（28.7） |
| **Intensive phase completed; maintenance phase unfinished** | 24（14.0） |
| **Incompleted induction-phase therapy** | 39（22.8） |

**Supplementary Table 9.** **Completion status of HD-IFN treatment duration.**
